# Atresia of ovarian follicles in fishes, and implications and uses in aquaculture and fisheries

**DOI:** 10.1111/jfd.13469

**Published:** 2021-06-16

**Authors:** Aldo Corriero, Rosa Zupa, Constantinos C. Mylonas, Letizia Passantino

**Affiliations:** ^1^ Department of Emergency and Organ Transplantation Section of Veterinary Clinics and Animal Production University of Bari Aldo Moro Valenzano (BA) Italy; ^2^ Institute of Marine Biology, Biotechnology and Aquaculture Hellenic Center for Marine Research Crete Greece

**Keywords:** oogenesis, ovary, oocytes, atretic follicles, teleosts

## Abstract

Atresia of ovarian follicles, that is the degenerative process of germ cells and their associated somatic cells, is a complex process involving apoptosis, autophagy and heterophagy. Follicular atresia is a normal component of fish oogenesis and it is observed throughout the ovarian cycle, although it is more frequent in regressing ovaries during the postspawning period. An increased occurrence of follicular atresia above physiological rates reduces fish fecundity and even causes reproductive failure in both wild and captive‐reared fish stocks, and hence, this phenomenon has a wide range of implications in applied sciences such as fisheries and aquaculture. The present article reviews the available literature on both basic and applied traits of oocyte loss by atresia, including its morpho‐physiological aspects and factors that cause a supraphysiological increase of follicular atresia. Finally, the review presents the use of early follicular atresia identification in the selection process of induced spawning in aquaculture and the implications of follicular atresia in fisheries management.

## 1 INTRODUCTION

Death of female germ cells during ovarian development and oogenesis is a phenomenon known to scientists since the 19th century (Flemming, 1885). It is an evolutionarily conserved event that involves invertebrates and vertebrates (Saidapur, 1978), including non‐human mammals and humans (Krysko et al., 2008; Matova & Cooley, 2001). Follicular atresia, that is the degenerative process of germ cells and their associated somatic cells, is a complex process involving apoptosis, a programmed form of cell death whose mechanisms are highly conserved in vertebrates and invertebrates, and are characterized by biochemical and structural changes, including chromatin condensation, DNA fragmentation, and formation of apoptotic bodies (Chen & Abrams, 2000; Corriero, Desantis, et al., 2007; Gross et al., 1999; Matova & Cooley, 2001; Metzstein et al., 1998; Vaux & Korsmeyer, 1999).

In the ovary of the adult nematode *Caenorhabditis elegans* Maupas, 1900, more than 50% of the germ cells are removed by apoptosis (Gumienny et al., 1999). Oocyte cell death is triggered by specific somatic cells, the so‐called sheath cells, that act as both germ cell death promoters and dead germ cell phagocytes (Li et al., 2012). In the fruit fly *Drosophila melanogaster* Meigen, 1830, each cystoblast undergoes four consecutive mitotic divisions to give rise to a cyst of 16 germ cells, one of which develops as an oocyte and the others differentiate into nurse cells, which provide nutrients to the growing oocytes. Germ‐cell death occurs in two different phases: during the first phase of yolk uptake by oocytes and after the final phase of cytoplasm transport from the nurse cells to oocytes that concludes with nurse cell death (Matova & Cooley, 2001).

Atresia has been described at all developmental stages of ovarian follicles in cyclostomes, in the ovaries of oviparous, ovoviviparous and viviparous elasmobranchs, as well as in unyolked and yolked ovarian follicles of Teleostei and Chondrostei (Linares‐Casenave et al., 2002; Mccully Phillips & Ellis, 2015; Saidapur, 1978; Waltrick et al., 2017). Follicular atresia of teleost fishes has been described as a complex process comprising apoptosis, autophagy—a catabolic process involved in the turnover of long‐lived proteins and organelles—and heterophagy, that is phagocytosis of egg components by granulosa cells acting as macrophages (Cassel et al., 2017; Santos et al., 2008; Thomé et al., 2009).

Ovarian follicle atresia has been documented also in amphibians: in the frog *Xenopus laevis* (Daudin, 1802), atresia affects mainly oocytes during the phase of yolk uptake (Saidapur, 1978 and references therein cited; Matova & Cooley, 2001). In reptiles, follicular atresia affects all stages of follicle development of many species (Saidapur, 1978 and references therein cited), among which the gecko *Hemidactylus mabouia* (Moreau de Jonnès, 1818) (Moodley & Van Wyk, 2007), the lizard *Sceloporus aeneus* Wiegmann, 1828 (Guillette & Jones, 1985) and the American alligator *Alligator mississippiensis* (Daudin, 1801) whose atretic follicles are particularly persistent and discernible with ovarian ultrasonography (Lance et al., 2009). Follicular atresia is largely documented in the adult ovary of many avian species and morphologically resembles the process described in reptiles (Saidapur, 1978 and references therein cited). In chick embryo, follicular atresia affects much more the right ovary, eventually destined to total regression (Ukeshima & Fujimoto, 1991). In mammals, atresia is a process of degeneration that affects ovarian follicles during perinatal life and postnatal life (Chun & Hsueh, 1998; Foghi et al., 1998; Krysko et al., 2008; Quirk et al., 2004; Tilly, 1996a). In antral follicles, the atretic process starts at the level of follicular/thecal cells and then it involves the oocyte; on the contrary, the atretic process seems to directly affect oocytes of primordial/primary follicles (Depalo et al., 2003; Tilly, 1996a). In humans, the ovarian follicle reserve is established during foetal life when around two‐thirds of oocytes are depleted via apoptosis. Subsequently, during adult life, this ovarian follicle reserve is gradually reduced by programmed cell death of the granulosa cells of the growing follicles (Hussein, 2005).

The aim of the present review was to summarize the existing literature on ovarian follicle atresia in fishes, with emphasis on its morphological aspects, physiological mechanisms and implications in aquaculture and fisheries management.

## 2 MORPHOLOGICAL ASPECTS OF ATRESIA

Morphological aspects of follicular atresia are similar in fish species. A widely accepted classification of atretic follicles is the four‐stage scheme proposed by Hunter and Macewicz (1985) for the northern anchovy *Engraulis mordax* Girard, 1854. The following description is based on the latter study and includes also personal observations of the authors. The iconography provided in the present review includes authors’ original micrographs of histological sections from greater amberjack *Seriola dumerili* (Risso, 1810), Atlantic bluefin tuna *Thunnus thynnus* (Linnaeus, 1758) and swordfish *Xiphias gladius* Linnaeus, 1758 ovaries. Unlike Hunter and Macewicz (1985), who used the term “atretic oocyte” to refer to the alpha (α) stage of atresia and the term “atretic follicle” to refer to the following stages (β, γ and δ), in the present review the term atretic follicle is used throughout, because the atretic process always involves follicular cells.

### 2.1 Alpha (α) stage

**FIGURE 1:**
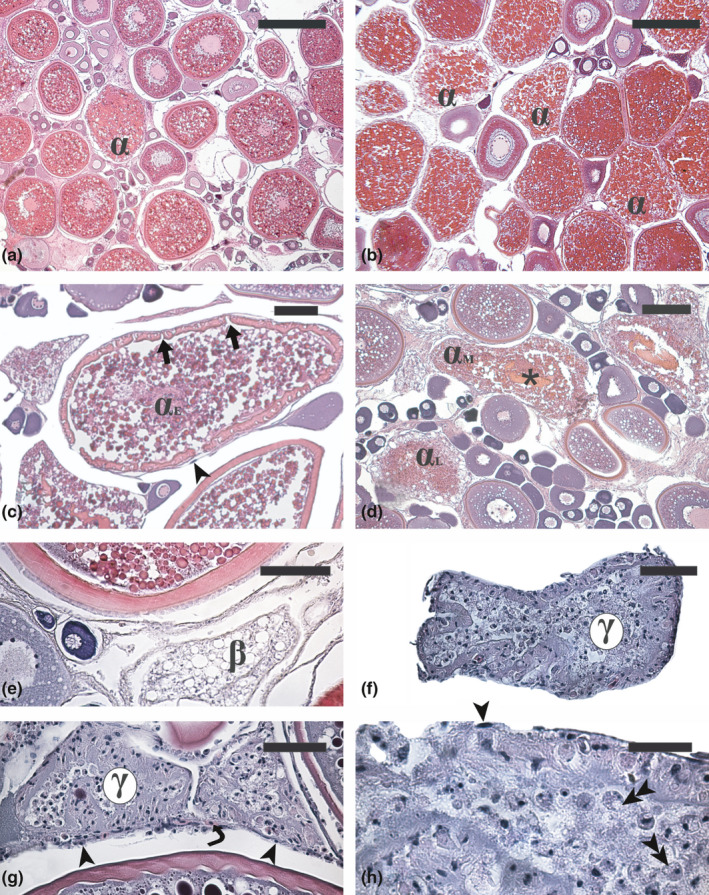
Micrographs of ovary sections from adult Atlantic bluefin tuna *Thunnus thynnus* (a, b, f, g and h), swordfish *Xiphias gladius* (e) and greater amberjack *Seriola dumerili* (c and d) in different phases of the reproductive cycle. (a) Advanced vitellogenic ovary showing a physiological rate of atresia. (b) Extensive atresia of vitellogenic follicles in a specimen that underwent an acute stressing event described in Corriero et al. (2011). (c) Early α‐atretic vitellogenic follicle (α_E_) characterized by zona radiata fragmentation and nucleus disappearance. (d) Mid (α_M_) and late (α_L_) atresia of vitellogenic follicles characterized by progressive zona radiata digestion and yolk granule coalescence. (e) β‐atretic follicle characterized by numerous lipid vesicles and total reabsorption of yolk granules. (f) Early and (g) late γ atretic follicles showing a progressive reduction of the number of follicular cells. (h) Particular of a late γ‐atretic follicle showing follicular cells in active phagocytosis. Haematoxylin‐eosin staining. Magnification bars: 400 µm in (a) and (b); 100 µm in (c) and (e); 150 µm in (d); 50 µm in (f) and (g); 30 µm in (h). α, α‐atretic vitellogenic follicle; β, β‐atretic follicle; γ, γ‐atretic follicle; arrow, zona radiata breakdown; arrowhead, thecal cell; double arrowhead, follicular cell in active phagocytosis; asterisk, residual zona radiata under digestion; curved arrow, blood vessel

In yolked oocytes, the first visible event of atresia is the lysis of the nuclear envelope followed by the dispersion of the nuclear content in the cytoplasm (Figure 1a,b). The oocyte becomes irregular in shape and yolk granules, and lipid droplets start to coalesce under the action of hydrolytic enzymes as indicated by the presence of fused or expanded globules (Figure 1b). Disappearance of striations due to oocyte microvilli withdrawal, progressive interruptions and loss of its thickness uniformity characterizes the rapid dissolution of the egg envelope (Figure 1c). In follicles at this stage of atresia, a small fraction of follicular cells degenerates by apoptosis (up to 10% in curimatã‐pacu *Prochilodus argenteus* Spix & Agassiz, 1829 and piau‐jejo *Leporinus taeniatus* Lütken, 1875; Santos et al., 2008). Following zona radiata fragmentation and breakdown, the invasion of enlarged granulosa cells into the oocyte points out the beginning of the second important event in α atretic follicles. Yolk granules liquefy, appearing as a uniform eosinophilic area in the oocyte cytoplasm (Figure 1d); yolk is then phagocytized by the granulosa cells. In this phase, blood vessels proliferate in the thecal layer of the follicles (Hunter & Macewicz, 1985).

### 2.2 Beta (β) stage

Following the complete degradation of the oocyte, the follicle enters the β stage of atresia (Figure 1e). At the beginning of this stage, follicular cells appear disorganized, some of them showing pyknotic nuclei and others containing intracellular, apparently empty vacuoles or vacuoles filled by amorphous material. A thin layer of thecal cells and blood vessels surrounds the follicular cells. In species whose oocytes contain abundant lipid droplets, numerous residual spherical vacuoles are distributed throughout the β atretic follicles (Hunter et al., 1986) (Figure 1e). At the end of the β stage, the atretic follicle may be involved in one of the three following patterns of degeneration: (i) the atretic follicle may progress through the following stages of atresia, the gamma (γ) and the delta (δ) stages; (ii) the atretic follicle can completely be reabsorbed at β stage without any further remaining structure; (iii) the atretic follicle can directly progress in the δ stage by missing out the γ phase. In late β atretic follicles, extensive apoptosis of follicular cells occurs (see below) (Morais et al., 2012). A large transient cavity has been sometime observed inside β atretic follicles of the killifish *Millerichthys robustus* (Miller & Hubbs 1974) (Dominguez‐Castanedo et al., 2019), which likely results from the extraction of a large lipid drop during tissue processing for histological analysis.

### 2.3 Gamma (γ) stage

The atretic follicle at the γ stage is smaller than the β stage follicle, and the granulosa cells are characterized by the presence of light‐yellow flocculent material in the cytoplasm as well as by irregular shape of the nuclei (Figure 1f,g). Phagocytosis of oocyte components by follicular cells is still active (Figure 1h). The number of theca cells and blood vessels that surround the granulosa cells in the γ stage is strongly reduced.

### 2.4 Delta (δ) stage

**FIGURE 2:**
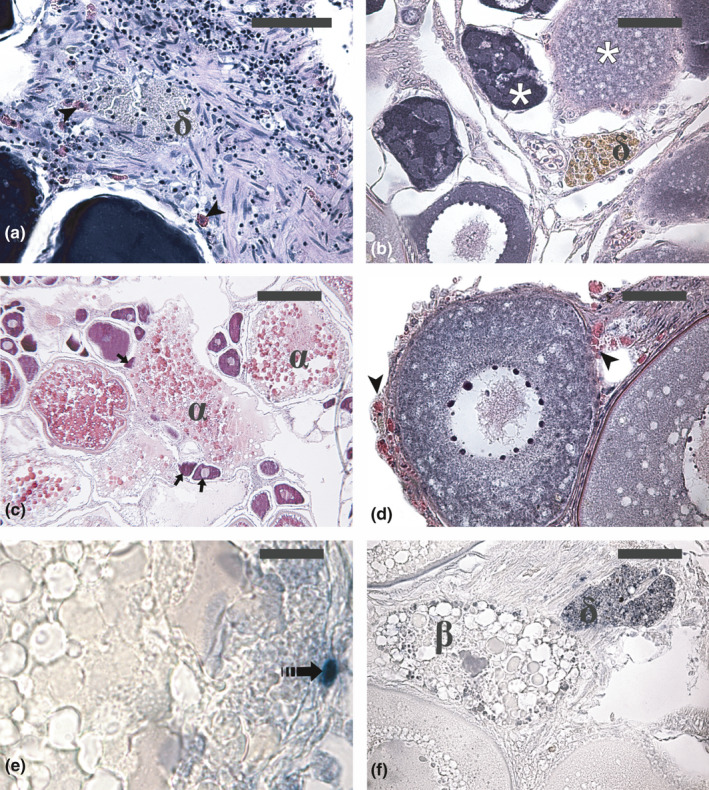
Micrographs of ovary sections from adult Atlantic bluefin tuna *Thunnus thynnus* (a, c, e and f) and swordfish *Xiphias gladius* (b and d) in different phases of the reproductive cycle. (a) δ‐atretic follicle showing yellow‐pigmented granules. (b) δ‐atretic follicle showing cells containing brownish pigments. (c) Degenerating unyolked follicles incorporated by an atretic vitellogenic follicle (arrow). (d) Eosinophilic granulocytes at the periphery of an early atretic previtellogenic follicle (arrowhead). (e) Apoptotic granulosa cell in an early α‐atretic vitellogenic follicle (dashed arrow). (f) Apoptotic cells and bodies (dark dots) in β and δ atretic follicles. Haematoxylin‐eosin staining in (a–d). Staining of apoptotic cells and bodies by the terminal deoxynucleotidyl transferase‐mediated 2′‐deoxyuridine 5′‐triphosphate nick end labelling (TUNEL) method in (e) and (f). Magnification bars: 50 µm in (a), b, d and f; 200 µm in (c) and 20 µm in (e). α, α‐atretic vitellogenic follicle; β, β‐atretic follicle; ẟ, ẟ‐atretic follicle; asterisk, atretic unyolked follicle

In δ stage atretic follicles, granulosa cells are drastically reduced in number and they contain yellow and/or brownish pigments (lipofuscins and melanin) whose appearance shows species‐specific characteristics (Figure 2a,b). The autofluorescence properties of lipofuscin make atretic follicles at this stage easily recognizable from the surrounding connective stroma in unstained histological sections under florescence microscopy (Medina et al., 2021). If follicular cells accumulate melanin, δ atretic follicles can become morphologically similar to melanomacrophage centres (Figure 2b). At the end of the atretic process, granulosa cells are no longer surrounded by thecal cells and blood vessels (Figure 2a).

Although atresia mainly affects follicles containing yolked oocytes, the occurrence of unyolked atretic follicles has been reported in many fish species (Corriero et al., 2003; Hunter & Macewicz, 1985; Miranda et al., 1999). Atresia affects previtellogenic oocytes at the perinucleolar and lipid‐cortical alveoli stage (Figure 2b) and unyolked oocytes at the initial stage of atresia show similar morphological characteristics to α atretic yolked oocytes but without yolk (Hunter & Macewicz, 1985). Further stages of atresia of unyolked oocytes have not been described because they are morphologically undistinguishable from advanced stages of atretic vitellogenic follicles.

Ultrastructural aspects of follicular atresia have been described by Miranda et al. (1999) in the freshwater fish species *Astyanax bimaculatus lacustris* (Lütken, 1875) and *Leporinus reinhardti* (Lütken, 1875). The electron microscopy observations provided details on the events that characterize early atretic degeneration: disintegration of the nuclear envelope and dispersion of chromatin in the ooplasm during early atresia; disintegration of organelles and cytoplasmic inclusions (mitochondria, annulate lamellae, cortical alveoli, yolk globules); fragmentation of the zona radiata; progressive convolution; and fragmentation of the basal membrane. Miranda et al. (1999) observed oocyte death by necrosis during the initial stages of atresia and apoptotic degeneration of follicular cells during the regression of the atretic follicle. These observations corroborate the assumption that the first event of the atretic process in fish, that is oocyte degeneration, is not mediated by apoptosis.

The biological meaning of the atresia of vitellogenic oocytes is related to the recovery of highly energetic moieties, whereas that of unyolked follicles is not clear. A possible explanation is that oocytes at advanced stages of primary growth that have not been recruited into vitellogenesis, once they have finalized the building of the organelle machinery and the synthesis of the membrane receptors needed for the uptake of exogenous proteins, cannot survive until the following reproductive season. In the daily spawner killifish, atresia affects both primary and secondary growth follicles from the onset of sexual maturity until fish death, supporting the hypothesis that atresia plays a role in selecting follicles able to perform vitellogenesis and subsequent stages of development (Dominguez‐Castanedo et al., 2019).

As described above, oocyte atresia involves the fragmentation of the oocyte envelope to allow follicular cells to enter the oocyte and act as macrophages (Corriero et al., 2004; Domínguez‐Castanedo et al., 2019; Linares‐Casenave et al., 2002; Passantino et al., 2020; Santos et al., 2008). The loss of integrity of the oocyte results in the spreading of its components—including lysosomal enzymes—outside the follicle and invasion of neighbouring structures (Figure 2c), an event that can lead to degradation of neighbouring structures, including unyolked and yolked follicles. This event, which may be classified as necrosis, explains the frequent finding of degenerating unyolked follicles near and sometimes inside atretic vitellogenic follicles (see below).

Eosinophilic granulocytes (Figure 2d) have been repeatedly reported in ovaries containing degenerating follicles (Besseau & Faliex, 1989; Bruslè‐Sicard & Fourcault, 1997; Chaves‐Pozo et al., 2003; De Metrio et al., 2003; Kokokiris et al., 1999), thus raising a possible role of these white blood cells in follicular atresia. In Atlantic bluefin tuna, eosinophilic granulocytes have been observed in the interstitial tissue of ovigerous lamellae as well as at the periphery of unyolked oocytes undergoing atresia (Corriero et al., 2003). Eosinophilic granulocytes have been observed in association with degenerating oocytes in swordfish intersex gonads (macroscopically male gonads with interspersed female germ cells) (De Metrio et al., 2003). In gilthead seabream *Sparus aurata* Linnaeus, 1758, acidophilic granulocytes showed high phagocytic activity towards bacteria and their morphological and functional characteristics have led to the consideration of this cell type as functionally equivalent to the neutrophils of higher vertebrates (Sepulcre et al., 2002). However, the accumulation of inteleukin‐1β in their cytoplasm prompted the hypothesis of a role related to the regulation of germ cell growth and/or steroidogenesis rather than to phagocytosis of degenerating germ cells (Chaves‐Pozo et al., 2003). Although the mechanisms mediating the invasion of immune cells in teleost atretic follicles are not known, Tingaud‐Sequeira et al. (2006) reported a high level of two transcripts encoding for chemotactic factors in Senegalese sole *Solea senegalensis* Kaup, 1858, atretic follicles: a transcript related to mammalian lect2, which encodes a protein with chemotactic properties for human neutrophils, and a transcript encoding the protein S100a10, a chemoattractant for leukocytes or activator of macrophages. This finding provides evidence for the presence of a chemotaxin‐mediated mechanism for leukocyte accumulation in fish atretic follicles, similarly to that occurring during the formation of the corpus luteum in the mammalian ovary (Townson & Liptak, 2003).

A peculiar mechanism of oocyte degeneration has been described in rainbow trout *Oncorhynchus mykiss* (Walbaum, 1792) by Schulz and Blüm (1983). This process consists of the extrusion of the nucleus, together with a portion of ooplasm, through an opening of the follicle. Once expelled from the oocyte, the nucleus reaches the ovarian lumen where it degenerates, or it is eventually discharged outside through the genital pore. According to the authors’ observation, this kind of degeneration involved all the advanced previtellogenic oocytes of a fraction of fish that were sampled during or after the reproductive season. The authors hypothesized that this peculiar kind of massive degeneration affected oocyte batches that were too late in the development to be recruited into vitellogenesis and maturation within the current reproductive cycle and could not survive until the next reproductive season.

## 3 MECHANISMS AND HORMONAL REGULATION OF ATRESIA

In mammals, the main molecular mechanism responsible for ovarian follicular atresia is apoptotic cell death (Hughes & Gorospe, 1991; Tilly et al., 1991). Apoptosis or programmed cell death is an evolutionarily conserved physiological process involved in tissue remodelling, differentiation and degeneration in a variety of cell types (Steller, 1995). There is considerable information concerning the intracellular pathways involved in ovarian apoptotic cell death in mammals as well as the role of many regulatory genes (Hughes & Gorospe, 1991; Tilly, 1996a, 1996b,1996a, 1996b; Tilly et al., 1997), among which those encoding proteins of the Bcl‐2 family that act as both “survival” and “death” factors (Hsu & Hsueh, 2000). However, in more recent times, there has been increasing evidence that apoptosis is not the exclusive mechanism and that autophagy represents an alternate form of programmed cell death responsible for follicular atresia both in invertebrates and vertebrates, including mammals (Duerrschmidt et al., 2006; Krysko et al., 2008).

The role of apoptosis in follicular atresia in teleosts has been studied by Wood and Van Der Kraak (2001), who found low level of DNA fragmentation during the early phase of atresia in rainbow trout and goldfish *Carassius auratus* (Linnaeus, 1758) vitellogenic follicles, thus excluding the possibility that apoptosis is the triggering event of follicular atresia. In early α atretic vitellogenic follicles of Atlantic bluefin tuna, the occurrence of apoptotic granulosa cells is a sporadic finding (author's unpublished data; Figure 2e). Through the immunohistochemical detection of proteins involved in the two forms of programmed cell death in the ovaries of three characiform species, Morais et al. (2012) showed that autophagy, a catabolic process involved in the turnover of long‐lived proteins and organelles, and apoptosis are activated in a coordinated fashion. Moreover, the co‐localization pattern of proteins involved in autophagy (cathepsin‐D and Beclin‐1) and apoptosis (caspase‐3, bax, bcl‐2) led the authors to propose that these proteins interplay in the mechanism of follicular atresia, which would represent the result of a complex interaction among these factors. According to the proposed model, autophagy is the prevailing event during early atresia; during the late stage of atresia, once they have finalized their phagocytotic activity towards oocyte yolk and organelles, follicular cells die by apoptosis. In late atretic follicles of Atlantic bluefin tuna, extensive apoptosis of follicular cells has been observed (authors’ unpublished data; Figure 2f). Therefore, the atretic process in teleost fishes involves at least three different processes (Santos et al., 2008): autophagy (self‐digestion of oocyte and follicular cell components), heterophagy (phagocytosis of egg components by granulosa cells that act as macrophages) and follicular cell death by apoptosis. Moreover, the breakdown of the zona radiata and the consequent release of hydrolytic enzymes in the extracellular space may lead to the necrotic death of neighbouring cells. The necrosis of ovarian tissue in proximity of atretic follicles (Figure 2c) does not make part of the atretic process itself; however, it may play an important role during the process of tissue renewal that follows the dramatic events related to ovulation (authors’ personal observation).

Although follicular cell apoptosis is part of the mechanism of follicular atresia in teleosts, the survival factor S100a10 (a protein that play an anti‐apoptotic role by binding the Bcl‐xL/Bcl‐2‐associated death promoter) is upregulated in Senegalese sole ovarian follicles undergoing atresia (Townson & Liptak, 2003). This apparent contradictory finding might be related to the need of assuring survival of follicular cells during the initial phases of atresia.

During vitellogenesis, oocytes of oviparous vertebrates accumulate large amounts of yolk proteins derived from the cleavage of vitellogenin, a phospholipoprotein synthesized in the liver (Hara et al., 2016; Patiño & Sullivan, 2002; Pousis et al., 2012, 2019). The fate of the yolk, which is massively reabsorbed during the atretic process, is not fully elucidated. It has been proposed that yolk‐derived proteins are hydrolysed in situ to free amino acids by lysosomal cathepsins (Wood & Van der Kraak, 2002). However, there is also clear evidence of a massive transfer of yolk proteins in the bloodstream in the course of follicular atresia (Babin, 1987a, 1987b,1987a, 1987b). The latter mechanism of yolk resorption has been confirmed by Tingaud‐Sequeira et al. (2006) who found that the genes apoa1 and apoc1, which encode for proteins making part of chylomicrons, very low‐density lipoproteins (VLDL) and high‐density lipoproteins (HDL) involved in lipid transportation in the bloodstream, are upregulated in atretic ovaries of Senegalese sole. The presence of egg yolk proteins in the plasma likely results in their rapid catabolism in organs other than the ovary, such as liver and kidney, which are the two main organs involved in the degradation of high‐density lipoproteins in rats (Pittman & Steinherg, 1984). In the liver of greater amberjack captured from the wild and reared in captivity, undergoing a reproductive dysfunction resulting in the extensive atresia of vitellogenic oocytes, high densities of melanomacrophage centres, which are involved in the destruction of endogenous and exogenous material, and apoptotic cells were observed, and this finding was correlated to the hepatic overload related to the metabolism of large amounts of yolk‐derived moieties (Passantino et al., 2020).

The role of the reproductive hormones in follicular atresia is not fully understood, and most of the knowledge on this issue comes from studies on the effects of various types of stressors on oogenesis (see §4). In mammals, it is well known that gonadotropins (GtH) and sex steroid hormones act as survival factors for germ cells and their withdrawal induce apoptosis (Young & Nelson, 2001). In Atlantic bluefin tuna reared in captivity, the systemic administration of a gonadotropin‐releasing hormone agonist (GnRHa) (Mylonas et al., 2007) resulted in a reduction of male germ cell loss by apoptosis (Corriero et al., 2009). This effect was supposed to be mediated by 11‐ketotestosterone (11‐KT), whose increased secretion was in turn attributed to a GnRHa‐induced luteinizing hormone (LH) release from the pituitary (Rosenfeld et al., 2012). In vitro experiments have shown that androgens act as survival factors for previtellogenic ovarian follicles of coho salmon *Oncorhynchus kisutch* (Walbaum, 1792). Forsgren and Young (2012) showed that 11‐KT but not 17β‐oestradiol (E_2_) stimulates the increase in size of late perinucleolar‐stage follicle. The use of an androgen receptor antagonist inhibited the growth‐promoting effect of 11‐KT and induced follicular atresia. Treatment with 11‐KT showed only a weak growth‐stimulating effect on oocytes at the following stage (cortical alveoli) of development, whose growth was instead stimulated by E_2_. In addition to E_2_, salmon gonadotropin (SG‐G100) and epidermal growth factor protected cultured rainbow trout follicular cells from apoptosis (Janz & Van der Kraak, 1997; Wood & Van der Kraak, 2002).

The liver synthesis of the egg yolk precursor vitellogenin is stimulated by E_2_, and there is evidence both in vitro (Talbott et al., 2011) and in vivo (Clearwater & Pankhurst, 1997; Coward et al., 1998; Mylonas et al., 1998, 2010; Zupa, Rodríguez, et al., 2017) that abnormally low E_2_ plasma concentrations are associated with a diminished capacity of oocyte to complete vitellogenesis, resume meiosis and undergo final maturation, and finally induce follicular atresia (see also § 4).

A few experimental studies suggest that a major pro‐apoptotic role is played by GnRH synthesized in the ovary and acting on follicular cells through an autocrine/paracrine mechanism. In goldfish ovary, GnRH was found to protect ovarian follicles in mid‐vitellogenesis from atresia, an effect that was not mediated by GtH (and then exerted through a local action). However, in mature, preovulatory follicles, exposure to GnRH induced follicular apoptosis, a pro‐apoptotic effect that was blocked by GtH (Habibi & Andreu‐Vieyra, 2007). Based on these data, as well as on experimental data on the effect of the co‐exposure to GnRH and GtH on oocyte meiosis resumption, Habibi and Andreu‐Vieyra (2007) proposed a model in which the fate of each ovarian follicle depends on the local ratio of GtH/GnRH. According to this model, ovarian follicles are destined to undergo atresia under the autocrine/paracrine action of GnRH; however, this GnRH action cannot be exerted if proper GtH concentrations are present, that is if ovulation is properly stimulated by a suitable preovulatory GtH surge.

As in mammals, the regulation of atresia in fish may also involve the control of angiogenesis. To this regard, Tingaud‐Sequeira et al. (2006) reported that, during follicular atresia of Senegalese sole, angiogenesis may be inhibited through the upregulation of the anti‐angiogenic factor thrombospondin isoform thbs.

## 4 FACTORS INDUCING FOLLICULAR ATRESIA

As reported above, follicular atresia is a normal component of fish oogenesis and it is observed throughout the ovarian cycle, although it is more frequent in regressing ovaries during the postspawning period (Ganias et al., 2008; Miranda et al., 1999; Saidapur, 1978). Generally, follicular atresia does not preclude the reproductive success of fish populations; however, an increase of the atretic rate beyond physiological rates can reduce the annual fecundity and even cause reproduction failure of both wild (Ganias et al., 2008; Hunter et al., 1992; Jørgensen et al., 2006; Kraus et al., 2008; Kurita et al., 2003; Neves et al., 2009; Rideout et al., 2005, Rideout and Tomkiewicz, 2011; Witthames & Greer Walker, 1995) and captive‐reared fish stocks (Corriero, Desantis, et al., 2007; Corriero, Medina, et al., 2007; Corriero et al., in press; Fakriadis, Miccoli, et al., 2020; Kjesbu et al., 1991; Ma et al., 1998; Mylonas et al., 2010; Pousis et al., 2018, 2019; Zupa et al., 2013; Zupa, Fauvel, et al., 2017; Zupa, Rodríguez, et al., 2017). Extensive follicular atresia and spawning omission are the most efficient strategy to optimize fecundity in case of low body energy reserves (Kennedy et al., 2008; Rideout et al., 2000, 2005) and, in general, the lower the food intake the higher the proportion of atretic vitellogenic follicles (Kennedy et al., 2008; Kjesbu et al., 1991; Ma et al., 1998; Scott, 1962). Incidentally, in farmed gilthead seabream, extensive atresia has been found to be associated to an inhibition of ovarian response to isotocin, a hormone that plays an important role in ovulation, oviduct contraction and spawning (Piccinno et al., 2014), suggesting that spawning is totally inhibited in ovaries with a high rate of atretic follicles.

Factors that have been shown to increase follicular atresia above physiological rates include food availability and energy reserves (Corriero et al., 2011; Hunter & Macewicz, 1985; Jørgensen et al., 2006; Kennedy et al., 2008; Skjæraasen et al., 2013), environmental and social factors (Rideout et al., 2005; Rideout & Tomkiewicz, 2011) and various other kind of stress (Schreck et al., 2001). The stress response depends on fish species, stage of maturity, and type and severity of stressor (Pankhurst & Van Der Kraak, 1997; Schreck et al., 2001). Impairment of reproductive performances, including reduction of egg production via atresia, is a common phenomenon associated with stress in fish (Barton & Iwama, 1991; Donaldson, 1990). Decrease in fecundity or spawning omission is caused by the energy reallocation occurring when fish experience a stressing event, which, therefore, does not affect only the anabolic processes of the individual fish but also its investment of energy in progeny (Wendelaar Bonga, 1997).

Corticosteroids, chiefly cortisol and catecholamines are the main mediators of fish response to acute stress (Schreck, 2010). Corticosteroids have a suppressive activity on hypothalamus, pituitary and gonad functions (reviewed by Schreck, 2010), and the effect of stress on the reproductive axis depends on the phase of the life cycle during which it has been experienced as well as on the nature of stressors, in terms of intensity and duration (i.e. acute or chronic stress) (Schreck, 2010). Stressors inducing follicular atresia through a cortisol‐mediated mechanism include confinement of wild fish in captivity, crowding and handling, as well as alteration of natural environmental parameters (temperature and photoperiod) (Clearwater & Pankhurst, 1997; Contreras‐Sánchez et al., 1998; Kjesbu et al., 1991; Schreck et al., 2001).

The deleterious cortisol‐mediated effects of stress on vitellogenesis, which eventually result in follicular atresia, are well documented in salmonids. Brown trout *Salmo trutta* Linnaeus, 1758, undergoing crowding‐induced stress, showed elevated plasma levels of adrenocorticotropic hormone and cortisol and decreased levels of circulating testosterone (T) and 11‐KT (Pickering et al., 1987). Experiments carried out in the rainbow trout demonstrated that cortisol injection during advanced vitellogenesis induces a significant short‐term reduction of plasma T and E_2_, but not of maturational GtH (i.e. LH) (Pankhurst & Van Der Kraak, 2000). This effect was not observed when cortisol was administered during early vitellogenesis or oocyte maturation, thus indicating that the action exerted by cortisol is stage‐specific and it likely involves GtH signal transduction but not LH pituitary secretion. Female brook trout *Salvelinus fontinalis* (Mitchill, 1814) exposed to acid stress showed lower vitellogenin levels (Roy et al., 1990), and cortisol implantation suppressed sex steroid synthesis and vitellogenesis in rainbow trout and brown trout (Carragher et al., 1989). It was proposed that the deleterious effects of cortisol on vitellogenesis are due to a transcriptional interference on the expression of liver E_2_ receptors, rather than on E_2_ secretion (Lethimonier et al., 2000).

Castranova et al. (2005) demonstrated that striped bass *Morone saxatilis* (Walbaum, 1792) that had genetically determined low cortisol responses to stressors, had lower androgen levels and lower spermiation response to gonadotropin treatment when subjected to stressors, which indicates that the response of the reproductive axis to stress is not only mediated by corticosteroids. The effects of environmental pollutants on fish reproduction are often mediated by toxic interactions with the endocrine control mechanisms of reproduction (Flouriot et al., 1995; Goksøyr & Förlin, 1992; McKinney & Waller, 1994). The mechanism with by starvation, one of the most powerful triggers of follicular atresia, interfere with the activity of the reproductive axis is not fully elucidated; however, it seems to involve growth hormone (Sumpter et al., 1991), insulin‐like growth factor 1, ovarian follicle stimulating hormone (FSH) receptors and pro‐apoptotic factors (Yamamoto et al., 2011), but not cortisol.

### 4.1 Starvation and crowding

Energy reserve availability, that is optimum food intake, is a prerequisite for the production of high‐quality gametes. In both wild and rearing environments (Mylonas et al., 2010; Rideout et al., 2000, 2005), an insufficient food intake is a well‐known trigger of follicular atresia. When food supply is insufficient, fishes adopt species‐specific strategies to increase survival chance, including reduction of fecundity through follicular atresia and spawning omission (Rideout et al., 2005; Rideout & Tomkiewicz, 2011; Schreck, 2010; Schreck et al., 2001).

In northern anchovy starved experimentally to elicit follicular atresia, vitellogenic oocytes were reabsorbed at a remarkable rate (46% of females showing α atretic yolked oocytes on the 3rd day after the onset of starvation) and the reintroduction of a normal feeding regime was followed by a rapid retrieval of normal oogenesis (Hunter & Macewicz, 1985). Wild Atlantic cod *Gadus morhua* Linnaeus, 1758, with low body and liver conditions showed extensive atresia of vitellogenic oocytes, suggesting that they might have experienced starvation (Rideout et al., 2000). Atlantic bluefin tuna caught by a tuna trap and therein starved up to 15 days showed a degree of atresia of vitellogenic follicles (36 to 100%) likely correlated to the number of days of starvation (Corriero et al., 2011). On the contrary, when gilthead seabream were fasted for many weeks during the spawning period, there was no negative effect on spawning performance (fecundity and fertilization success), egg proximate composition or embryo and early larval development (Mylonas CC, unpublished data). Apparently, this species, which has a very long reproductive period (3–5 months) and produces annually more the 2.5 times its body weight in eggs, is able to deal with long periods of starvation and maternal nutrient reserves were mobilized to maintain optimal egg nutrient composition (Mylonas et al., 2011).

Fasting as a stress inducer has been shown to exacerbate the negative effects of crowding in zebrafish *Danio rerio* (Hamilton, 1822). In this species, acute (3 hr) and chronic (5 days) crowding resulted in a fourfold increase in whole‐body cortisol level, and this increase was further triggered by starvation, suggesting an interaction between the two stressing factors in stimulating fish corticosteroid response (Ramsay et al., 2006). Fish are very sensitive to crowding, and a sudden increase of fish density has been often used in experiments aimed at increasing our understanding of fish response to stress (Barton, 2002; Castranova et al., 2005; Wendelaar Bonga, 1997); however, the knowledge of crowding on follicular atresia is very limited. Substrate‐spawning redbelly tilapia *Tilapia zillii* (Gervais, 1848) undergoing crowding showed a dramatic reduction of E_2_ and T plasma levels and oogenesis arrest followed by follicular atresia. When normal rearing conditions were restored, both steroids rapidly rose to original levels and oogenesis restarted (Coward et al., 1998). It was suggested that the reduced levels of E_2_ and T during crowding were insufficient to allow completion of vitellogenic growth and were responsible for oogenesis arrest and atresia. The mechanism leading to E_2_ and T suppression has not been elucidated; however, crowding factors, that is pheromones released in the water from fish reared at uncomfortable high density, have been proposed as possible mediators of the observed inhibition of reproductive axis activity (Coward et al., 1998; Pfuderer et al., 1974). Crowding factors have been supposed to exert an inhibitory effect on growth and reproduction and to depress heart rate (Pfuderer et al., 1974). Sex steroids made hydrophilic through glucuronide and sulphate conjugation and excreted to the water through fish urine, free steroids released through the gills (Zohar, 2021 and references therein cited) as well as prostaglandin F2α (Mylonas et al., 2017) are well‐known pheromones that facilitate successful reproduction and spawning by synchronizing reproductive activity. However, to our knowledge, the identity of purported pheromones acting as reproduction inhibitors in high‐density rearing conditions have not been discovered yet.

### 4.2 Environmental pollution

Pollution of aquatic environments involves a broad range of chemical compounds deriving from human activities (industrial, agricultural, food industries, pharmaceutical, discharges of domestic sewage effluents), many of which interfere with organism endocrine function by acting either as agonist or antagonist of hormones (Johnson et al., 1999). Chronic pollution of aquatic environments may affect fish reproductive success by decreasing the quality of gametes, thus inducing a significant risk for the survival of fish populations (Au, 2004).

Severe ovarian histopathological findings, including follicular atresia, were reported in wild pelagic and benthic species exposed to pesticides (Chukwuka et al., 2019), as well as in zebrafish experimentally exposed to non‐steroidal pharmaceuticals (Madureira et al., 2011) and synthetic progestin (Jiang et al., 2019), in fathead minnow *Pimephales promelas* Rafinesque, 1820, experimentally exposed to heavy metals (Driessnack et al., 2017a, 2017b,2017a, 2017b) and in climbing perch *Anabas testudineus* (Bloch, 1792) experimentally exposed to a pesticide (Mohapatra et al., 2020).

Studies carried out on fish captured from areas polluted by industrial and municipal spills showed that the onset of follicular atresia is usually associated with a decrease of E_2_ plasma concentration (Aguilar et al., 2007; Au, 2004; Janz et al., 1997; Jobling et al., 2002; Johnson et al., 1999; Mayon et al., 2006), suggesting that follicular atresia results from hypothalamic–pituitary–ovarian disruption, with inhibition of GTH release and consequent impairment of steroidogenesis. An inhibitory action of xenoestrogens on GtH release (negative feedback) and a consequent increase in follicular atresia has been reported in many fish species both in the wild (Agbohessi et al., 2015) and in experimental studies (Kaptaner & Ünal, 2011; Kiparissis et al., 2003; Mandich et al., 2007; Miles‐Richardson et al., 1999; Ye et al., 2014). Moreover, endocrine‐disrupting chemicals can directly induce follicular atresia by triggering cell death signalling. In fact, apoptosis of thecal and granulosa cells has been observed in fish exposed to pharmaceutical, industrial and municipal wastewater in both field (Janz et al., 1997; Prado et al., 2014) and experimental studies (Chen et al., 2016; Galus, Jeyaranjaan, et al., 2013; Galus, Kirischian, et al., 2013; Kaptaner & Ünal, 2011; Mishra & Mohanty, 2008, 2012, 2014), leading to hypothesize that pollutants might directly damage ovaries through the reduction of follicular cells available for steroid production. In zebrafish, sublethal dietary exposure to 2,3,7,8‐tetrachlorodibenzo‐p‐dioxin (TCDD) inhibited the transition from previtellogenesis to vitellogenesis, by affecting the capability of ovaries to synthetize E_2_ and thus reducing the stimulation of hepatic vitellogenin synthesis, eventually leading to follicular atresia (King Heiden et al., 2006).

Contrary to the above studies, white sucker *Catostomus commersoni* (Lacepède, 1803) living in water exposed to bleached kraft pulp mill effluent (Janz et al., 1997) as well as Guinean tilapia *Tilapia guineensis* (Günther, 1862) and African catfish *Clarias gariepinus* (Burchell, 1822) inhabiting water polluted by pesticides (Agbohessi et al., 2015) displayed follicular atresia associated with an increase in E_2_ plasma concentrations, and the hypothesis that high levels of E_2_ might be due to an upregulation of aromatase was proposed. In fact, transcription of genes encoding steroidogenic enzymes might be either up‐ or down‐regulated by exposure of fish to xenobiotics (Galus, Jeyaranjaan, et al., 2013; King Heiden et al., 2006; Molina et al., 2013, 2018; Sridevi et al., 2015; Wirbisky et al., 2016; Ye et al., 2014) and confinement‐induced stress (Zupa, Rodríguez, et al., 2017). In general, as suggested by Au (2004), the effect of environmental pollutants on E_2_ secretion and follicular atresia is species‐specific, depending on types of pollutants (e.g. metal or oestrogen‐mimetic substances), life traits (e.g. migratory or sedentary species), and reproductive state (e.g. before or during vitellogenesis).

The negative impact of anthropogenic activities on the aquatic environment is not only represented by the spillage of polluting substances, but also by the drastic alteration of a geographical area for industrial purposes. For instance, the deviation or damming of watercourses by the construction of dams alters the local ecological balance and causes changes in the chemical–physical parameters (mainly temperature and dissolved oxygen concentration). These affect the reproductive biology of the inhabiting fish species, by reducing sex steroid concentrations, inducing extensive follicular atresia and decreasing fecundity (Agostinho et al., 1993; Arantes et al., 2010, 2011; Perini et al., 2013; Sato et al., 2005; Thomé et al., 2012). Low dissolved oxygen concentration also characterizes the so‐called coastal “dead zones,” that is hypoxic zones mainly occurring in the northern hemisphere and originating from the combined action of natural phenomena such as upwelling and anthropogenic fertilization of marine systems by excess nitrogen (Diaz & Rosenberg, 2008). An increased incidence of follicular atresia and reduced fecundity has been observed in Atlantic croaker *Micropogonias undulatus* (Linnaeus, 1766) living in a hypoxic area in the northern Gulf of Mexico (Thomas et al., 2005), and it was experimentally demonstrated that atresia was associated to follicular cell apoptosis triggered by hypoxia (Ondricek & Thomas, 2018).

Finally, recent studies suggest that also exposure to microplastics (Yan et al., 2020) and air pollution (Sayed et al., 2018) through an increase in UVR penetrating marine ecosystem might impair fish reproductive function and cause follicular atresia.

### 4.3 Temperature and photoperiod

Whatever the habitat is—marine, freshwater or brackish—water temperature is one of the most effective environmental factors in triggering oocyte maturation and spawning in fish of the temperate zones (Billard et al., 1981; Bromage et al., 2001; Mylonas et al., 2010; Rideout et al., 2005). Natural or artificially induced abnormal changes in water temperatures can cause severe stress and lead to alteration of fish homeostasis (Schreck, 2010; Schreck et al., 2001), and when environmental conditions suitable for offspring survival are lost, extensive follicular atresia may occur in mature ovaries of both migrating and sedentary fish (Schreck et al., 2001).

Experiments conducted in Chondrostei (white sturgeon *Acipenser transmontanus* Richardson, 1836, Russian sturgeon *A. gueldenstaedtii* Brandt & Ratzeburg, 1833 and stellate sturgeon *A. stellatus* Pallas, 1771) showed that exposure to high and constant temperature (about 18℃) induced a decrease in sex steroids and ovarian degeneration via follicular atresia (Dettlaff & Daydova, 1979; Dettlaff et al., 1993; Kazanskii, 1963; Linares‐Casenave et al., 2002; Webb et al., 1999; Webb et al., 2001). In the white sturgeon, a decrease in sex steroids (T and E_2_) and vitellogenin plasma levels, followed by extensive follicular atresia, occurred in individuals experimentally exposed to warm water (18–20℃) when the maturational competence (i.e. 100% follicles at germinal vesicle breakdown stage) had not yet been reached, but not in fish that had already attained the maturational competence (Linares‐Casenave et al., 2002). These results suggested the hypothesis of a temperature‐sensitive phase in sturgeon ovarian development that might coincide with the transition from vitellogenic growth to maturational competence, which is a gonadotropin‐dependent step (Dettlaff et al., 1993). The increase of water temperature, caused by discharge from nuclear power plants in Sweden and Lithuania, was observed to affect oogenesis and seriously compromise reproductive potential also in perch *Perca fluviatilis* Linnaeus, 1758, roach *Rutilus rutilus* (Linnaeus, 1758) and pike *Esox lucius* Linnaeus, 1758 (Lukšienė et al., 2000).

A sudden decrease in water temperature may also induce follicular atresia. Modifications in local current pattern of Barents Sea was considered the main cause of vitellogenic oocyte resorption in Greenland halibut *Reinhardtius hippoglossoides* (Walbaum, 1792) (Fedorov, 1971). The upheaval of local environmental conditions, including water temperature decrease, caused by the construction of the Três Marias Dam (Brazil), induced an extensive resorption of vitellogenic follicles in curimatã‐pacu inhabiting the São Francisco River close to the dam (Arantes et al., 2010; Sato et al., 2005). An episode of northern Atlantic cod mass atresia and skipped spawning, recorded in 1999 in Smith Sound, Newfoundland, was attributed to the co‐occurrence of unusual low temperature (0–0.5 vs 3–4℃) and reduced food availability (Rideout et al., 2000).

The role of photoperiod in regulating the timing of reproduction in teleost fish is well known (Zohar et al., 2010) and, according to their photoperiod preferences, fish may be classified into long‐day type and short‐day types (Yoshioka, 1962, 1963). However, in the natural environment photoperiod varies in parallel with temperature, so that the effects of abrupt changes of each of the two variables on fish reproductive capacity are difficult to be discerned (Billard et al., 1981; Clark et al., 2005; Koger et al., 1999).

In aquaculture practices, daylight modulation (i.e. short‐ or long‐day lengths) is commonly applied in order to advance or delay gonad maturation and ovulation (Bromage et al., 2001; Mylonas et al., 2010). Nevertheless, if not properly regulated with respect to the physiological responsiveness to light stimuli, photoperiod can also impair the normal gametogenesis process of a fish species. Reduction in photoperiod (i.e. short‐day length exposure) induced oocyte regression via follicular atresia in the long‐day type species medaka *Oryzias latipes* (Temminck & Schlegel, 1846) (Koger et al., 1999; Yoshioka, 1963). Follicular atresia was experimentally induced in the freshwater fish spotted snakehead *Channa punctatus* (Bloch, 1793) by means of treatments with melatonin, the hormone secreted by pineal organ under environmental photoperiodic stimuli that takes part to the regulation of reproductive cycles (Bromage et al., 1995, 2001; Falcón et al., 2010), designed to simulate prolonged darkness (Renuka & Joshi, 2010). Continuous artificial lighting accelerates oocyte reabsorption in Atlantic salmon *Salmo salar* Linnaeus, 1758, and it is a useful tool to recondition fish and enhance flesh quality and increase fish commercial value (Porter et al., 2003).

## 5 ASPECTS OF ATRESIA IN CAPTIVITY AND HORMONAL INDUCTION OF SPAWNING

When reared in captivity, almost all fishes exhibit some reproductive dysfunction, ranging from complete lack of gametogenesis to lack of oocyte maturation, ovulation and spawning (Mylonas et al., 2010). Complete lack of gametogenesis is rare, and among the fishes produced currently under aquaculture conditions, it is limited to freshwater eels (genus *Anguilla*) (da Silva et al., 2018). In most captive‐reared fishes, oocytes enter vitellogenesis, but depending on the rearing or welfare conditions, females (a) may undergo extensive atresia towards the end of vitellogenesis or (b) may fail to undergo oocyte maturation. In both cases, fish fail to ovulate and spawn.

**FIGURE 3:**
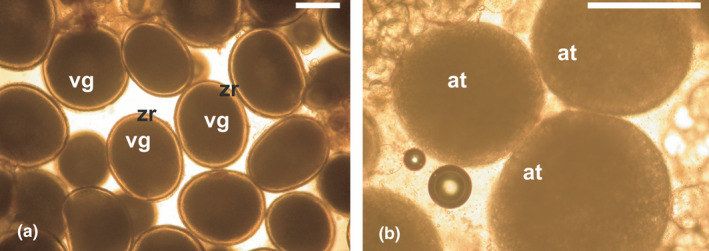
Wet mounts from ovarian biopsies of greater amberjack *Seriola dumerili* sampled at the onset of the spawning season. (a) Fully vitellogenic oocytes (vg) with dark ooplasm and distinct zona radiata (zr). (b) Vitellogenic oocytes undergoing advanced follicular atresia (at) having a lighter ooplasm and no apparent zr. Magnification bars = 500 μm

In the first case, where females undergo extensive atresia towards the end of vitellogenesis, no corrective measures can be taken when the situation is identified. Efforts must be made to improve rearing conditions for the next reproductive cycle, such as improving photothermal conditions, reducing stocking density and increasing tank size, improving water quality or improving broodstock nutrition. For example, greater amberjack maintained in land‐based tanks supplied with bore‐hole sea water, as opposed to surface sea water, often undergo extensive atresia towards the end of vitellogenesis, therefore they do not reliably reach the stage of being eligible for hormonal induction of maturation, ovulation and spawning (Fakriadis, Sigelaki, et al., 2020). An ovarian biopsy of such females exhibits a large percentage of vitellogenic oocytes undergoing follicular atresia, which is visible not only after histological processing, but also in a wet mount under 100 or 40x magnification. In wet mounts, vitellogenic oocytes undergoing advanced follicular atresia can be distinguished from healthy fully vitellogenic oocytes based on (a) the absence of the zona radiata and (b) a lightening of the cytoplasm (Figure 3a,b). Females undergoing such extensive follicular atresia will not reproduce and will not respond to any hormonal spawning induction therapy, and should be discarded from this year's spawning population.

**FIGURE 4:**
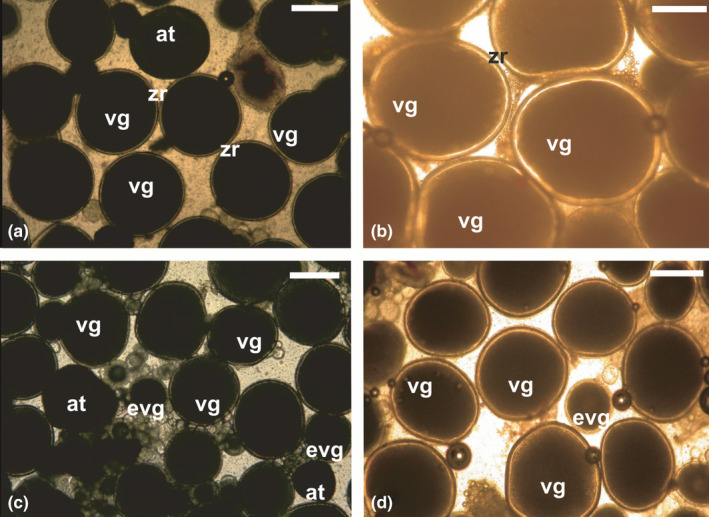
Wet mounts from ovarian biopsies from (a) European sea bass *Dicentrarchus labrax*, (b) wreckfish *Polyprion americanus,* (c) meagre *Argyrosomus regius* (d) greater amberjack *Seriola dumerili* at the onset of the spawning season, showing fully vitellogenic oocytes (vg) with a distinct zona radiata (zr) and some sparse oocytes in follicular atresia (at), as well as oocytes in early vitellogenesis (evg). Magnification bars = 500 μm

In the second case of reproductive failure in captivity, females complete vitellogenesis but fail to undergo oocyte maturation, and thus ovulation and spawning. To induce maturation, females are commonly treated with exogenous hormones, such as gonadotropin preparations (carp pituitary extracts, CPE; human chorionic gonadotropin, hCG or recombinant luteinizing hormone, rLH) or synthetic GnRH agonists (GnRHa) with or without dopamine antagonists (Mañanos et al., 2009; Mylonas et al., 2010; Zohar & Mylonas, 2001). The fish are then allowed to spawn volitionally in tanks, or in species that also fail to spawn in captivity—such as salmonids, carps and some flatfishes—their eggs are obtained manually by stripping and they are fertilized artificially. In this situation, eligible females are selected based on the mean oocyte diameter of their largest vitellogenic oocytes and the absence of extensive follicular atresia. To respond to the hormonal treatment, females must have completed vitellogenesis, and, therefore, reached the maximum size of vitellogenic oocytes. In species with synchronous ovarian development, the mean diameter of a random sample of vitellogenic oocytes is considered, whereas in fishes with asynchronous or group synchronous ovarian development the mean diameter of the largest oocytes is considered (Mañanos et al., 2009). For example, in the synchronous spawner striped bass, the mean diameter of the postvitellogenic oocytes is 800 μm (Mylonas et al., 1997) and it tends to increase as the fish grow in size. In the group‐synchronous European sea bass *Dicentrarchus labrax* (Linnaeus, 1758) and wreckfish *Polyprion americanus* (Bloch & Schneider, 1801), the diameter of the fully vitellogenic oocytes is 700 μm (Mylonas et al., 2003) and 1200 μm (Papadaki et al., 2018), respectively (Figure 4a and b). Finally, in the asynchronous meagre *Argyrosomus regius* (Asso, 1801) and greater amberjack, the mean diameter of the fully vitellogenic oocytes is 550 μm (Duncan et al., 2018) and 600 μm (Fakriadis, Miccoli, et al., 2020), respectively (Figure 4c and d).

**FIGURE 5:**
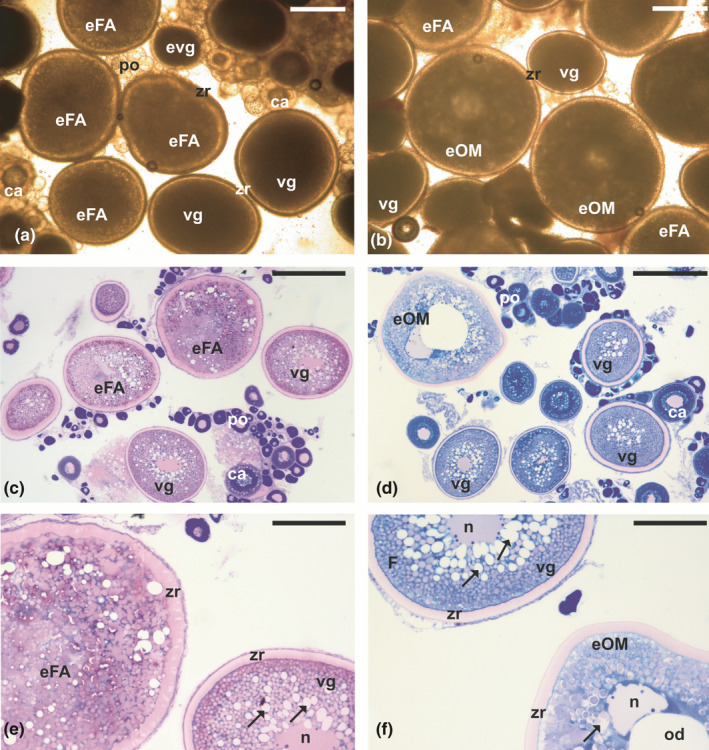
Wet mounts from ovarian biopsies from different greater amberjack *Seriola dumerili* breeders at the onset of the spawning season, showing (a) oocytes at the very early stage of follicular atresia (eFA), and (b) oocytes undergoing early maturation (eOM). Vitellogenic oocytes (vg), as well as primary oocytes (po) and cortical alveoli oocytes (ca) may also exist in the biopsies. Histological sections from the same ovarian biopsies show vitellogenic and eFA oocytes (c and e), the latter exhibiting zona radiata (zr) enlargement and fragmentation, and ooplasm disorganization; and eOM oocytes (d and f) showing lipid droplet (arrows) coalescence into eventually a single oil droplet (od), while the germinal vesicle (nucleus, n) is still intact and the yolk globules still dispersed. Magnification bars =500 μm (a, d) and 200 μm (e, f)

In addition to evaluating the oocyte diameter, the occurrence of follicular atresia is also considered, and a small number of atretic oocyte (<2% in the viewed wet mount) is not considered a problem. In fact, it is often a desirable characteristic, since it is thought to confirm that vitellogenesis has reached its end, and from now on unless induced to mature, the oocytes will begin to undergo atresia when water temperatures increase above the optimal limits (Fakriadis, Miccoli, et al., 2020; Mylonas et al., 2013). Finally, the appearance of (a) a distinct zona radiata, appearing as a bright “halo” around the oocyte, and (b) a uniformly dark ooplasm in these postvitellogenic oocytes are also considered prerequisites of eligibility for hormonal induction of maturation. These latter two characteristics are extremely useful to identify the very early signs of follicular atresia (eFA), which may often be confused with early oocyte maturation. The eFA oocytes may be slightly larger than normal postvitellogenic oocytes, and although they also have a uniform ooplasm and a visible zona radiata, they are lighter in colour, slightly translucent and appear to have a thicker zona radiata (Figure 5a). These oocytes may be confused by the untrained observer with oocytes undergoing early maturation (eOM), a process associated with marked increases in diameter due to hydration, together with lipid droplet coalescence and localized clearing of the cytoplasm (Mylonas et al., 1997). A more careful examination of eFA oocytes identifies a number of differences from eOM oocytes (Figure 5b). For example, the zona radiata in eOM oocytes does not become much thicker than postvitellogenic oocytes, and it is very distinct; in eFA oocytes, the zona radiata becomes thicker (2×) and appears more diffuse. Also, in eOM oocytes the ooplasm also becomes lighter in colour, but this “clearing” is localized and not uniform. This is caused by the coalescence of the lipid droplets into eventually a single or few “oil droplet(s)” located in the centre of the egg, a process that is very common in the buoyant pelagic eggs of most marine fishes (Mylonas et al., 1997).

Under histological evaluation, it becomes evident that these eFA oocytes do not undergo lipid coalescence, their cytoplasm is disorganized and the enlarged zona radiata begins to fragment (Figure 5c and e). Another significant event is that the germinal vesicle (or nucleus) is no longer visible in the centre of the oocyte, where it was throughout the process of vitellogenesis. Nuclear dissolution and DNA breakdown, and zona radiata fragmentation are the very first morphological signs of apoptosis and follicular atresia (Miranda et al., 1999). On the other hand, histological examination of oocytes that are undergoing eOM confirms that the zona radiata does not become thicker and is not fragmented, the germinal vesicle is still intact and may begin its migration to the periphery, while the lipid droplets are clearly coalescing to form larger droplets (Figure 5d and f).

Based on experience from a number of different marine fishes, females that have a large occurrence of such eFA oocytes are not good candidates for hormonal spawning induction (Mylonas CC, personal observations). Such females may undergo oocyte maturation, ovulation and spawning, but very often with low fecundity and poor egg quality. Presumably, this is because the eFA oocytes do not undergo maturation ‐so a smaller number of eggs is produced. Furthermore, even the ones that appear normal postvitellogenic oocytes and do undergo oocyte maturation, apparently they are somehow compromised and produce eggs of poor quality. Poor quality means that fertilization success may be low, and the embryonic development and survival are also reduced. So, being able to detect the very early onset of follicular atresia in aquaculture fishes is very important when implementing hormonal spawning induction therapies, in fishes that present reproductive dysfunctions in captivity or when implementing breeding selection programmes with in vitro fertilization.

## 6 ECOLOGICAL ASPECTS AND IMPACT OF ATRESIA ON FISH REPRODUCTIVE POTENTIAL

Atresia plays a significant role in fish reproductive strategies, as it is a fine‐tuning mechanism by which a species regulates fecundity (Brown‐Peterson et al., 2011; Kennedy et al., 2008; Rideout et al., 2000, 2005). According to Brown‐Peterson et al. (2011), fish reproductive strategies can be classified based on spawning pattern (total or partial spawners) and type of fecundity (determinate or indeterminate). Total spawners (referred to earlier as of the synchronous ovarian type) are fish species that spawn a single batch during the annual reproductive season. Total spawners have a determinate fecundity since their oocytes develop synchronously and are then released at once. Partial or batch spawners spawn multiple batches of oocytes, and can display either a determinate or an indeterminate fecundity. In partial spawners with determinate fecundity (group synchronous ovarian type), two oocyte populations can be distinguished at a given time, a synchronously developing population of larger oocytes, which will be eventually released in successive batches, and a heterogeneous population of smaller oocytes representing the following group‐synchronous oocyte populations. In partial spawners with indeterminate fecundity (asynchronous ovarian type), oocytes are continuously recruited into vitellogenesis without a dominant population (Brown‐Peterson et al., 2011; Murua et al., 2003; Wallace & Selman, 1981).

In both total and partial spawners with determinate fecundity, the amount of atretic follicles in the regression phase is limited. In the total spawners Baltic cod *Gadus morhua callarias* Linnaeus, 1758, and black scabbardfish *Aphanopus carbo* Lowe, 1839, follicular atresia usually occurs at low levels in the prespawning and spawning phase, as a mechanism to both regulate the number of eggs that will be spawned and remove damaged or abnormal oocytes (Bromley et al., 2000; Kraus et al., 2008; Neves et al., 2009). In partial spawners with indeterminate fecundity, atresia of vitellogenic oocytes occurs throughout the reproductive season and it becomes marked after the spawning phase, during ovarian regression, that is when the remaining vitellogenic oocytes are reabsorbed (Brown‐Peterson et al., 2011; Pérez & Figuiredo, 1992;).

Food availability and optimal fish body condition are prerequisites for success in reproduction; however, the way by which females regulate fecundity when food availability is limited is species specific (Rideout et al., 2005). Indeed, when food is scarce, fishes can adopt two different strategies to recover energy and assure survival: some species reabsorb vitellogenic oocytes via atresia (Hislop et al., 1978; Ma et al., 1998; Scott, 1962), and other species regulate fecundity by limiting the number of oocytes that are recruited into vitellogenesis (Bagenal, 1969; Burton, 1994; Horwood et al., 1989; Tyler & Dunn, 1976). The strategy of energy retrieval may depend also on the phase of the reproductive cycle in which food access is restricted (Rideout et al., 2005).

Atresia is a potential source of error for fecundity estimates of a fish population, and an incorrect evaluation of its rate can make fisheries management measures ineffective (Armstrong & Whittames, 2004; Cooper et al., 2005; Hunter et al., 1992; Neves et al., 2009). The main problems in the evaluation of the effect of atresia on reproductive potential are both the short duration of atretic stages, which makes their detection in histological samples problematic (Hunter & Macewicz, 1985), and the difficulty in the identification of advanced atretic stages, so that usually only the first (alpha) stage of atresia is taken into consideration for quantification (Murua at al., 2003). In fish species with determinate fecundity, potential annual fecundity (i.e. the total number of advanced vitellogenic oocytes matured per female and year, uncorrected for oocytes lost by atresia) is already determined before spawning takes place (Hunter et al., 1992; Kjesbu et al., 1991; Kraus et al., 2008; Murua et al., 2003; Óskarsson et al., 2002; Plaza et al., 2007; Thorsen et al., 2006). For these species, the actual (realized) fecundity is currently estimated as the potential annual fecundity minus the number of oocytes degenerated by atresia (Armstrong & Whittames, 2004; Brown‐Peterson et al., 2011; Murua et al., 2003). In total spawner, realized annual fecundity is usually assessed in prespawning individuals in late vitellogenesis, whereas in partial spawners with determinate fecundity it is usually assessed before the release of the first oocyte batch (Armstrong & Whittames, 2004; Brown‐Peterson et al., 2011; Murua et al., 2003; Neves et al., 2009). In partial spawners with indeterminate fecundity, in which the potential annual fecundity is not predetermined, total annual fecundity is calculated as batch fecundity (number of eggs spawned during each spawning event) multiplied by the estimated number of spawning events (Murua et al., 2003). Batch fecundity estimation is commonly carried out during the spawning capable phase and takes into account ovaries containing oocytes at maturation stage, hydrated oocytes or postovulatory follicles (Armstrong & Whittames, 2004; Brown‐Peterson et al., 2011; Hunter & Macewicz, 1985; Murua et al., 2003), so there is no need to correct for eggs lost via atresia in this case (Murua et al., 2003).

Despite the relevance of oocyte loss via atresia in determining fecundity, studies quantifying the difference between potential and realized fecundity are limited. In studies carried out on wild fish populations, estimated oocyte loss by atresia was around 8% in Dover sole *Microstomus pacificus* (Lockington, 1879) caught in the Pacific (Hunter et al., 1992) and between 12.4% and 30.5% in common sole *Solea solea* (Linnaeus, 1758) from the Atlantic (Horwood, 1993; Witthames & Greer Walker, 1995). Atretic degeneration was reported to involve 6 to 13% of western mackerel *Scomber scombrus* Linnaeus, 1758 oocytes (Greer Walker et al., 1994) and 35 to 55% of spring‐spawning herring *Clupea harengus* Linnaeus, 1758 oocytes (Óskarsson et al., 2002). Extensive down‐regulation of realized fecundity compared to potential fecundity was observed in turbot *Scophthalmus maximus* (Linnaeus, 1758) in the Baltic Sea (Nissling et al., 2016). On the contrary, atresia did not significantly affect fecundity in bluemouth *Helicolenus dactylopterus dactylopterus* (Delaroche, 1809) caught in the north‐western Mediterranean; indeed, a low percentage (1.45%) of atretic oocytes was observed in the spawning‐capable phase of this species (Muñoz et al., 2010). Interestingly, a reduction of potential fecundity by 27% was reported for the Baltic cod during the spawning season 2000 (Kraus et al., 2008), whereas the incidence of atresia on fecundity in individuals captured during the spawning seasons 2015–2016 was much lower (8%), despite the population was experiencing a high stress, showing low body condition, disappearance of larger individuals and decrease in the mean length at sexual maturity, due to changes in environmental and ecological factors (Mion et al., 2018). These apparently contradictory results were explained by changes in fecundity regulation strategies of the stressed population, which “decided” to down regulate the number of oocytes entering vitellogenesis before the beginning of the spawning season instead of down regulate fecundity by reabsorbing oocyte via follicular atresia during the spawning phase (Mion et al., 2018).

Yolk reabsorption through extensive follicular atresia represents also one of the mechanisms by which fish populations or a group of individuals from a population, skip a reproductive season (Jørgensen et al., 2006; Neves et al., 2009; Rideout et al., 2005; Rideout & Tomkiewicz, 2011). Skipped spawning via oocyte reabsorbing can be caused by poor nutrition, but also by environmental factor such as low temperature and pollution (Rideout et al., 2005, and reference therein cited). In most cases, individuals that skip spawning via follicular atresia are young adults that approach puberty, but eventually address the available energy towards somatic growth rather than reproduction (Jørgensen et al., 2006; Rideout & Rose, 2006; Rideout et al., 2005; Rideout & Tomkiewicz, 2011).

The annual fraction of the population that omit spawning is considered in recruitment projections for hoki *Macruronus novaezelandiae* (Hector, 1871) (Livingston et al., 1997) and orange roughy *Hoplostethus* *atlanticus* Collett, 1889 (Bell et al., 1992) stocks in the south‐west Pacific Ocean. As regards Atlantic fish species, the population fractions that omit spawning are poorly known and seldom considered in fisheries models. However, it is widely reported that the Atlantic cod may not spawn annually once reaching maturation (Rideout et al., 2000 and references therein cited). Electronic tagging experiments and fishery data have shown that not all the adult individuals of the highly migratory species Atlantic bluefin tuna visit any of the known spawning grounds during the spawning season, thus leading to the disputed hypothesis that only a fraction of the adult population contributes to recruitment (Bello et al., 2021; Corriero et al., 2020; Medina, 2020), a reproductive strategy that could involve follicular atresia as mechanism underlying spawning omission.

## 7 CONCLUSIONS

Follicular atresia affects both previtellogenic and vitellogenic follicles; however, the knowledge regarding its morphological and physiological aspects concerns mostly vitellogenic follicles. Although follicular atresia is a natural physiological process, several factors have been shown to increase follicular atresia above normal rates, including fasting and various other kind of stress. Follicular atresia can be also induced by pollution of aquatic environments due to a broad range of chemical compounds deriving from human activities that interfere with endocrine functions by acting either as agonist or antagonist of hormones. Anthropogenic activities may be responsible of changes of the aquatic environment, such as changes in temperature and oxygen concentration, affecting the reproductive biology of the inhabiting fish species and inducing extensive follicular atresia.

In captive‐reared fishes, oocytes enter vitellogenesis but they may undergo extensive atresia towards the end of vitellogenesis or may fail to undergo oocyte maturation. Atresia of vitellogenic follicles can be diagnosed through the analysis of an ovarian wet‐mount biopsy. Spawning stock biomass is an important parameter in fisheries management and its estimation may be biased if atretic follicle rates are not considered in the calculation of fish fecundity. Significant fractions of fish populations may omit spawning due to the occurrence of extensive follicular atresia, and this should also be considered in the estimation of a fish stock reproductive potential.

The present analysis of the literature made evident that the knowledge on the mechanisms and hormonal regulation of atresia is still insufficient. Considering the severe implications of atresia on the reproduction of cultured fish, a more in‐depth knowledge of its hormonal control might help overcome reproduction dysfunctions and improve the existing technologies for reproduction control in aquaculture. The incidence of atretic rates must be carefully considered in wild fish populations exposed to aquatic pollution as it represents a sign of impairment of reproductive function and can reduce significantly fish stock reproductive potential. Finally, the systematic use of gonad histological analysis to calculate atretic rates would improve fecundity estimates and help refine fishery regulation measures of endangered fish stocks.

## CONFLICT OF INTEREST

The authors declare no competing or financial interests.

## References

[jfd13469-bib-0001] Agbohessi, P. T., Imorou Toko, I., Ouédraogo, A., Jauniaux, T., Mandiki, S. N. M., & Kestemont, P. (2015). Assessment of the health status of wild fish inhabiting a cotton basin heavily impacted by pesticides in Benin (West Africa). Science of the Total Environment, 506–507, 567–584. 10.1016/j.scitotenv.2014.11.047 25433386

[jfd13469-bib-0002] Agostinho, A. A., Mendes, V. P., Suzuki, H. T., & Canzi, C. (1993). Avaliação da atividade reprodutiva da comunidade de peixes dos primeiros quilômetros a jusante do reservatório de Itaipu. Revista Unimar, 15(Suppl), 175–189.

[jfd13469-bib-0003] Aguilar, C., González‐Sansón, G., Hernández, I., MacLatchy, D. L., & Munkittrick, K. R. (2007). Effects‐based assessment in a tropical coastal system: Status of bicolor damselfish (*Stegastes part*itus) on the north shore of Cuba. Ecotoxicology and Environmental Safety, 67, 459–471. 10.1016/j.ecoenv.2006.05.004 16857259

[jfd13469-bib-0004] Arantes, F. P., dos Santos, H. B. , Rizzo, E., Sato, Y., & Bazzoli, N. (2011). Collapse of the reproductive process of two migratory fish (*Prochilodus argenteus* and *Prochilodus costatus*) in the Três Marias Reservoir, São Francisco River, Brazil. Journal of Applied Ichthyology, 27, 847–853. 10.1111/j.1439-0426.2010.01583.x

[jfd13469-bib-0005] Arantes, F. P., Santos, H. B., Rizzo, E., Sato, Y., & Bazzoli, N. (2010). Profiles of sex steroids, fecundity, and spawning of the curimatã‐pacu *Prochilodus argenteus* in the São Francisco River, downstream from the Três Marias Dam, Southeastern Brazil. Animal Reproduction Science, 118, 330–336. 10.1016/j.anireprosci.2009.07.004 19683404

[jfd13469-bib-0006] Armstrong, M. J., & Witthames, P. R. (2004). Developments in understanding of fecundity of fish stocks in relation to egg production methods for estimating spawning stock biomass. Fisheries Research, 117–118, 35–47. 10.1016/j.fishres.2010.12.028

[jfd13469-bib-0007] Au, D. W. T. (2004). The application of histo‐cytopathological biomarkers in marine pollution monitoring: A review. Marine Pollution Bulletin, 48, 817–834. 10.1016/j.marpolbul.2004.02.032 15111029

[jfd13469-bib-0008] Babin, P. J. (1987a). Apolipoproteins and the association of egg yolk proteins with plasma high density lipoproteins after ovulation and follicular atresia in the rainbow trout (*Salmo gairdneri*). The Journal of Biological Chemistry, 262, 4290–4296. 10.1016/S0021-9258(18)61346-8 3470295

[jfd13469-bib-0009] Babin, P. J. (1987b). Plasma lipoprotein and apolipoprotein distribution as a function of density in the rainbow trout (*Salmo gairdneri*). Biochemical Journal, 246, 425–429. 10.1042/bj2460425 PMC1148292 3689318

[jfd13469-bib-0010] Bagenal, T. B. (1969). The relationship between food supply and fecundity in brown trout *Salmo trutta* L. Journal of Fish Biology, 1, 167–182. 10.1111/j.1095-8649.1969.tb03850.x

[jfd13469-bib-0011] Barton, B. A. (2002). Stress in fishes: A diversity of responses with particular reference to changes in circulating corticosteroids. Integrative and Comparative Biology, 42, 517–525. 10.1093/icb/42.3.517 21708747

[jfd13469-bib-0012] Barton, B. A., & Iwama, G. K. (1991). Physiological changes in fish from stress in aquaculture with emphasis on the responses and effects of corticosteroids. Annual Review of Fish Diseases, 1, 3–26. 10.1016/0959-8030(91)90019-G

[jfd13469-bib-0013] Bell, J. D., Lyle, J. M., Bulman, C. M., Graham, K. J., Newton, G. M., & Smith, D. C. (1992). Spatial variation in reproduction, and occurrence of non‐reproductive adults, in orange roughy, *Hoplostethus atlanticus* Collett (Trachichthyidae), from south‐eastern Australia. Journal of Fish Biology, 40, 107–122. 10.1111/j.1095-8649.1992.tb02558.x

[jfd13469-bib-0014] Bello, G., Santamaria, N., & Corriero, A. (2021). Multiple‐phase biometric relationships and sexual maturity in the Atlantic Bluefin Tuna, *Thunnus thynnus* (Osteichthyes: Scombridae). Animals, 11, 390. 10.3390/ani11020390 33546441 PMC7913654

[jfd13469-bib-0015] Besseau, L., & Faliex, E. (1989). Presence of granulocytes and brown bodies in the ovotestis of *Lithognathus mormyrus* (L.) (Teleost, Sparidae). Ichtyophysiologica Acta, 13, 109–114.

[jfd13469-bib-0016] Billard, R., Bry, C., & Gillet, C. (1981). Stress, environment and reproduction in teleost fish. In A. D. Pickering (Ed.), Stress and fish (pp. 185–208). Academic Press.

[jfd13469-bib-0017] Bromage, N. R., Porter, M. J. R., & Randal, C. F. (2001). The environmental regulation of maturation in farmed finfish with special reference to the role of photoperiod and melatonin. Aquaculture, 197, 63–98. 10.1016/S0044-8486(01)00583-X

[jfd13469-bib-0018] Bromage, N. R., Randall, C. F., & Porter, M. J. R. (1995). How do photoperiod, the pineal gland, melatonin and circannual rhythms interact to coordinate seasonal reproduction in salmonids? In F. Goetz, & P. Thomas (Eds.), Reproductive Physiology of Fish V Symposium (pp. 164–166). University of Texas Publications.

[jfd13469-bib-0019] Bromley, P. J., Ravier, C., & Witthames, P. R. (2000). The influence of feeding regime on sexual maturation, fecundity and atresia in first‐time spawning turbot. Journal of Fish Biology, 56, 264–278. 10.1111/j.1095-8649.2000.tb02105.x

[jfd13469-bib-0020] Brown‐Peterson, N. J., Wyanski, D. M., Saborido‐Rey, F., Macewicz, B. J., & Lowerre‐Barbieri, S. K. (2011). A standardized terminology for describing reproductive development in fishes. Marine and Coastal Fisheries: Dynamics, Management, and Ecosystem Science, 3, 52–70. 10.1080/19425120.2011.555724

[jfd13469-bib-0021] Bruslè‐Sicard, S., & Fourcault, B. (1997). Recognition of sex inverting protandric *Sparus aurata*: Ultrastructural aspects. Journal of Fish Biology, 50, 1094–1103. 10.1111/j.1095-8649.1997.tb01633.x

[jfd13469-bib-0022] Burton, M. P. M. (1994). A critical period for nutritional control of early gametogenesis in female winter flounder, *Pleuronectes americanus* (Pisces: Teleostei). Journal of Zoology, 233, 405–415. 10.1111/j.1469-7998.1994.tb05273.x

[jfd13469-bib-0023] Carragher, J. F., Sumfter, J. P., Pottinger, T. G., & Pickering, A. D. (1989). The deleterious effects of cortisol implantation on reproductive function in two species of trout, *Salmo trutta* L. and *Salmo gairdneri* Richardson. General and Comparative Endocrinology, 76, 310–321. 10.1016/0016-6480(89)90163-9 2591721

[jfd13469-bib-0024] Cassel, M., Camargo, M. P., Wender, O. L., & Borella, M. I. (2017). Involution processes of follicular atresia and post‐ovulatory complex in characid fish ovary: A study of apoptosis and autophagy pathways. Journal of Molecular Histology, 48, 243–257. 10.1007/s10735-017-9723-6 28455549

[jfd13469-bib-0025] Castranova, D. A., King, V. W., & Woods, L. C. III (2005). The effects off stress on androgen production, spermiation response and sperm quality in high and low cortisol responsive domesticated male striped bass. Aquaculture, 246, 413–422. 10.1016/j.aquaculture.2004.12.019

[jfd13469-bib-0027] Chaves‐Pozo, E., Pelegrín, P., Mulero, V., Meseguer, J., & García Ayala, A. (2003). A role for acidophilic granulocytes in the testis of the gilthead seabream (*Sparus aurata* L., Teleostei). Journal of Endocrinology, 179, 165–174. 10.1677/joe.0.1790165 14596668

[jfd13469-bib-0028] Chen, H., Cao, J., Li, L., Wu, X., Bi, R., Klerks, P. L., & Xie, L. (2016). Maternal transfer and reproductive effects of Cr (VI) in Japanese medaka (*Oryzias latipes*) under acute and chronic exposures. Aquatic Toxicology, 171, 59–68. 10.1016/j.aquatox.2015.12.011 26748265

[jfd13469-bib-0029] Chen, P., & Abrams, J. M. (2000). Drosophila apoptosis and Bcl‐2 genes: Outliers fly in. Journal of Cell Biology, 148, 625–627. 10.1083/jcb.148.4.625 PMC2169364 10684245

[jfd13469-bib-0030] Chukwuka, A., Ogbeide, O., & Uhunamure, G. (2019). Gonad pathology and intersex severity in pelagic (*Tilapia zilli*) and benthic (*Neochanna diversus* and *Clarias gariepinus*) species from a pesticide‐impacted agrarian catchment, south‐south Nigeria. Chemosphere, 225, 535–547. 10.1016/j.chemosphere.2019.03.073 30901648

[jfd13469-bib-0031] Chun, S. Y., & Hsueh, A. J. W. (1998). Paracrine mechanisms of ovarian follicle apoptosis. Journal of Reproductive Immunology, 39, 63–75. 10.1016/s0165-0378(98)00013-8 9786453

[jfd13469-bib-0032] Clark, R. W., Henderson‐Arzapalo, A., & Sullivan, C. V. (2005). Disparate effects of constant and annually‐cycling daylength and water temperature on reproductive maturation of striped bass (*Morone saxatilis*). Aquaculture, 249, 497–513. 10.1016/j.aquaculture.2005.04.001

[jfd13469-bib-0033] Clearwater, S. J., & Pankhurst, N. W. (1997). The response to capture and confinement stress of plasma cortisol, plasma sex steroids and vitellogenic oocytes in the marine teleost, red gurnard. Journal of Fish Biology, 50, 429–441. 10.1111/j.1095-8649.1997.tb01370.x

[jfd13469-bib-0034] Contreras‐Sánchez, W. M., Schreck, C. B., Fitzpatrick, M. S., & Pereira, C. B. (1998). Effects of stress on the reproductive performance of rainbow trout (*Oncorhynchus mykiss*). Biology of Reproduction, 58, 439–447. 10.1095/biolreprod58.2.439 9475400

[jfd13469-bib-0035] Cooper, D. W., Pearson, K. E., & Gunderson, D. R. (2005). Fecundity of shortspine thornyhead (*Sebastolobus alascanus*) and longspine thornyhead (*S. altivelis*) (Scorpaenidae) from the northeastern Pacific Ocean, determined by stereological and gravimetric techniques. Fishery Bulletin, 103, 15–22.

[jfd13469-bib-0036] Corriero, A., Acone, F., Desantis, S., Zubani, D., Deflorio, M., Ventriglia, G., Bridges, C. R., Labate, M., Palmieri, G., McAllister, B. G., Kime, D. E., & De Metrio, G. (2004). Histological and immunohistochemical investigation on ovarian development and plasma estradiol levels in the swordfish (*Xiphias gladius* L.). European Journal of Histochemistry, 48, 413–421. 10.4081/915 15718208

[jfd13469-bib-0037] Corriero, A., Desantis, S., Bridges, C. R., Kime, D. E., Megalofonou, P., Santamaria, N., Cirillo, F., Ventriglia, G., Di Summa, A., Deflorio, M., Campobasso, F., & De Metrio, G. (2007). Germ cell proliferation and apoptosis during different phases of swordfish (*Xiphias gladius* L.) spermatogenetic cycle. Journal of Fish Biology, 70, 83–99. 10.1111/j.1095-8649.2006.01257.x

[jfd13469-bib-0038] Corriero, A., Desantis, S., Deflorio, M., Acone, F., Bridges, C. R., De la Serna, J. M., Megalofonou, P., & De Metrio, G. (2003). Histological investigation on the ovarian cycle of the bluefin tuna in the western and central Mediterranean. Journal of Fish Biology, 63, 108–119. 10.1046/j.1095-8649.2003.00132.x

[jfd13469-bib-0039] Corriero, A., Heinisch, G., Rosenfeld, H., Katavić, I., Passantino, L., Zupa, R., Grubišić, L., & Lutcavage, M. E. (2020). Review of sexual maturity in Atlantic bluefin tuna, *Thunnus thynnus* (Linnaeus, 1758). Reviews in Fisheries Science and Aquaculture, 28, 182–192. 10.1080/23308249.2019.1685456

[jfd13469-bib-0040] Corriero, A., Medina, A., Mylonas, C. C., Abascal, F. J., Deflorio, M., Aragón, L., Bridges, C. R., Santamaria, N., Heinisch, G., Vassallo‐Agius, R., Belmonte, A., Fauvel, C., Garcia, A., Gordin, H., & De Metrio, G. (2007). Histological study of the effects of treatment with gonadotropin‐releasing hormone agonist (GnRHa) on the reproductive maturation of captive‐reared Atlantic bluefin tuna (*Thunnus thynnus* L.). Aquaculture, 272, 675–686. 10.1016/j.aquaculture.2007.08.030

[jfd13469-bib-0041] Corriero, A., Medina, A., Mylonas, C. C., Bridges, C. R., Santamaria, N., Deflorio, M., Losurdo, M., Zupa, R., Gordin, H., de la Gándara, F. , Belmonte Ríos, A., Pousis, C., & De Metrio, G. (2009). Proliferation and apoptosis of male germ cells in captive Atlantic bluefin tuna (*Thunnus thynnus* L.) treated with gonadotropin‐releasing hormone agonist (GnRHa). Animal Reproduction Science, 116, 346–357. 10.1016/j.anireprosci.2009.02.013 19304415

[jfd13469-bib-0042] Corriero, A., Wylie, M. J., Nyuji, M., Zupa, R., & Mylonas, C. C. (in press). Reproduction of greater amberjack (*Seriola dumerili*) and other members of the family Carangidae. Reviews in Aquaculture. 10.1111/raq.12544

[jfd13469-bib-0043] Corriero, A., Zupa, R., Bello, G., Mylonas, C. C., Deflorio, M., Genovese, S., Basilone, G., Buscaino, G., Buffa, G., Pousis, C., De Metrio, G., & Santamaria, N. (2011). Evidence that severe acute stress and starvation induce rapid atresia of vitellogenic follicles in Atlantic bluefin tuna, *Thunnus thynnus* (L.) (Osteichthyes: Scombridae). Journal of Fish Diseases, 34, 853–860. 10.1111/j.1365-2761.2011.01303.x 21988357

[jfd13469-bib-0044] Coward, K., Bromage, N. R., & Little, D. C. (1998). Inhibition of spawning and associated suppression of sex steroid levels during confinement in the substrate‐spawning *Tilapia zillii* . Journal of Fish Biology, 52, 152–165. 10.1111/j.1095-8649.1998.tb01560.x

[jfd13469-bib-0045] da Silva, F. F. G. , Jacobsen, C., Kjørsvik, E., Støttrup, J. G., & Tomkiewicz, J. (2018). Oocyte and egg quality indicators in European eel: Lipid droplet coalescence and fatty acid composition. Aquaculture, 496, 30–38. 10.1016/j.aquaculture.2018.07.008

[jfd13469-bib-0046] De Metrio, G., Corriero, A., Desantis, S., Zubani, D., Cirillo, F., Deflorio, M., Bridges, C. R., Eicker, J., de la Serna, J. M. , Megalofonou, P., & Kime, D. E. (2003). Evidence of a high percentage of intersex in the Mediterranean swordfish. Marine Pollution Bulletin, 46, 358–361. 10.1016/S0025-326X(02)00233-3 12604071

[jfd13469-bib-0047] Depalo, R., Nappi, L., Loverro, G., Bettocchi, S., Caruso, M. L., Valentini, A. M., & Selvaggi, L. (2003). Evidence of apoptosis in human primordial and primary follicles. Human Reproduction, 18, 2678–2682. 10.1093/humrep/deg507 14645191

[jfd13469-bib-0048] Dettlaff, T. A., & Davydova, S. I. (1979). Differential sensitivity of cells of follicular epithelium and oocytes in the stellate sturgeon to unfavorable conditions, and correlating influence of triiodothyronine. General and Comparative Endocrinology, 39, 236–243. 10.1016/0016-6480(79)90228-4 499751

[jfd13469-bib-0049] Dettlaff, T. A., Ginsburg, A. S., & Schmalhausen, O. I. (1993). Sturgeon fishes: Developmental biology and aquaculture. Springer Verlag.

[jfd13469-bib-0050] Diaz, R. J., & Rosenberg, R. (2008). Spreading dead zones and consequences for marine ecosystems. Science, 321(5891), 926–929. 10.1126/science.1156401 18703733

[jfd13469-bib-0051] Domínguez‐Castanedo, O., Uribe, M. C., & Muñoz‐Campos, T. M. (2019). Morphological patterns of cell death in ovarian follicles of primary and secondary growth and postovulatory follicle complex of the annual killifish *Millerichthys robustus* (Cyprinodontiformes: Cynolebiidae). Journal of Morphology, 280, 1668–1681. 10.1002/jmor.21056 31433075

[jfd13469-bib-0052] Donaldson, E. M. (1990). Reproductive indices as measures of the effects of environmental stressors in fish. American Fisheries Society Symposium, 8, 109–122.

[jfd13469-bib-0053] Driessnack, M. K., Jamwal, A., & Niyogi, S. (2017a). Effects of chronic exposure to waterborne copper and nickel in binary mixture on tissue‐specific metal accumulation and reproduction in fathead minnow (*Pimephales promelas*). Chemosphere, 185, 964–974. 10.1016/j.chemosphere.2017.07.100 28753743

[jfd13469-bib-0054] Driessnack, M. K., Jamwal, A., & Niyogi, S. (2017b). Effects of chronic waterborne cadmium and zinc interactions on tissue specific metal accumulation and reproduction in fathead minnow (*Pimephales promelas*). Ecotoxicology and Environmental Safety, 140, 65–75. 10.1016/j.ecoenv.2017.02.023 28235657

[jfd13469-bib-0055] Duerrschmidt, N., Zabirnyk, O., Nowicki, M., Ricken, A., Hmeidan, F. A., Blumenauer, V., Borlak, J., & Spanel‐Borowski, K. (2006). Lectin‐like oxidized low‐density lipoprotein receptor‐1‐mediated autophagy in human granulosa cells as an alternative of programmed cell death. Endocrinology, 147, 3851–3860. 10.1210/en.2006-0088 16690797

[jfd13469-bib-0056] Duncan, N. J., Mylonas, C. C., Milton Sullon, E., Karamanlidis, D., França Nogueira, M. C., Ibarra‐Zatarain, Z., Chiumento, M., & Aviles Carrillo, R. O. (2018). Paired spawning with male rotation of meagre *Argyrosomus regius* using GnRHa injections, as a method for producing multiple families for breeding selection programs. Aquaculture, 495, 506–512. 10.1016/j.aquaculture.2018.06.017

[jfd13469-bib-0057] Fakriadis, I., Miccoli, A., Karapanagiotis, S., Tsele, N., & Mylonas, C. C. (2020). Optimization of a GnRHa treatment for spawning commercially reared greater amberjack *Seriola dumerili*: Dose response and extent of the reproductive season. Aquaculture, 521, 735011. 10.1016/j.aquaculture.2020.735011

[jfd13469-bib-0058] Fakriadis, I., Sigelaki, I., Papadaki, M., Papandroulakis, N., Raftopoulos, A., Tsakoniti, K., & Mylonas, C. C. (2020). Control of reproduction of greater amberjack *Seriola dumerili* reared in aquaculture facilities. Aquaculture, 519, 734880. 10.1016/j.aquaculture.2019.734880

[jfd13469-bib-0059] Falcón, J., Migaud, H., Muñoz‐Cueto, J. A., & Carrillo, M. (2010). Current knowledge on the melatonin system in teleost fish. General and Comparative Endocrinology, 165, 469–482. 10.1016/j.ygcen.2009.04.026 19409900

[jfd13469-bib-0060] Fedorov, K. Y. (1971). The state of the gonads of the Barents Sea Greenland halibut [*Reinhardtius hippoglossoides* (Walb.)] in connection with failure to spawn. Journal of Ichthyology, 11, 673–682.

[jfd13469-bib-0061] Flemming, W. (1885). Über die Bildung von Richtungsfiguren in Säugethiereiern beim Untergang Graaf’scher Follikel. Arch Anat Entwickl, 488 pp.

[jfd13469-bib-0062] Flouriot, G., Pakdel, F., Ducouret, B., & Valotaire, Y. (1995). Influence of xenobiotics on rainbow trout liver estrogen receptor and vitellogenin gene expression. Journal of Molecular Endocrinology, 15, 143–151. 10.1677/jme.0.0150143 8800639

[jfd13469-bib-0063] Foghi, A., Teerds, K. J., van der Donk, H. , Moore, N. C., & Dorrington, J. (1998). Induction of apoptosis in thecal/interstitial cells: Action of transforming growth factor (TGF) á plus TGFâ on bcl‐2 and interleukin‐1â‐converting enzyme. Journal of Endocrinology, 157, 489–494. 10.1677/joe.0.1570489 9691982

[jfd13469-bib-0064] Forsgren, K. L., & Young, G. (2012). Stage‐specific effects of androgens and estradiol‐17beta on the development of late primary and early secondary ovarian follicles of coho salmon (*Oncorhynchus kisutch*) in vitro. Biology of Reproduction, 87(64), 1–14. 10.1095/biolreprod.111.098772 22674392

[jfd13469-bib-0065] Galus, M., Jeyaranjaan, J., Smith, E., Li, H., Metcalfe, C., & Wilson, J. Y. (2013). Chronic effects of exposure to a pharmaceutical mixture and municipal wastewater in zebrafish. Aquatic Toxicology, 132–133, 212–222. 10.1016/j.aquatox.2012.12.016 23351725

[jfd13469-bib-0066] Galus, M., Kirischian, N., Higgins, S., Purdy, J., Chow, J., Rangaranjan, S., Li, H., Metcalfe, C., & Wilson, J. Y. (2013). Chronic, low concentration exposure to pharmaceuticals impacts multiple organ systems in zebrafish. Aquatic Toxicology, 132–133, 200–211. 10.1016/j.aquatox.2012.12.021 23375851

[jfd13469-bib-0067] Ganias, K., Nunes, C., & Stratoudakis, Y. (2008). Use of late ovarian atresia in describing spawning history of sardine, *Sardina pilchardus* . Journal of Sea Research, 60, 297–302. 10.1016/j.seares.2008.06.004

[jfd13469-bib-0068] Goksøyr, A., & Förlin, L. (1992). The cytochrome P‐450 system in fish, aquatic toxicology and environmental monitoring. Aquatic Toxicology, 22, 287–312. 10.1016/0166-445X(92)90046-P

[jfd13469-bib-0069] Greer Walker, M., Witthames, P. R., De Los, B., & Santos, I. (1994). Is the fecundity of mackerel (*Scomber scombrus*: Scombridae) determinate? Sarsia, 79, 13–26. 10.1080/00364827.1994.10413543

[jfd13469-bib-0070] Gross, A., McDonnell, J. M., & Korsmeyer, S. J. (1999). BCL‐2 family members and the mitochondria in apoptosis. Genes & Development, 13, 1899–1911. 10.1101/gad.13.15.1899 10444588

[jfd13469-bib-0071] Guillette, L. J. Jr, & Jones, R. E. (1985). Ovarian, oviductal, and placental morphology of the reproductively bimodal lizard, *Sceloporus aeneus* . Journal of Morphology, 184, 85–98. 10.1002/jmor.1051840109 29991190

[jfd13469-bib-0072] Gumienny, T. L., Lambie, E., Hartwieg, E., Horvitz, H. R., & Hengartner, M. O. (1999). Genetic control of programmed cell death in the *Caenorhabditis elegans* hermaphrodite germline. Development, 126, 1011–1022. 10.1242/dev.126.5.1011 9927601

[jfd13469-bib-0073] Habibi, H. R., & Andreu‐Vieyra, C. V. (2007). Hormonal regulation of follicular atresia in teleost fish. In P. J. Babin, J. Cerdà, & E. Lubzens (Eds.), The fish oocyte: from basic studies to biotechnological applications (pp. 235–253). Springer.

[jfd13469-bib-0074] Hara, A., Hiramatsu, N., & Fujita, T. (2016). Vitellogenesis and choriogenesis in fishes. Fisheries Science, 82, 187–202. 10.1007/s12562-015-0957-5

[jfd13469-bib-0075] Hislop, J. R. G., Robb, A. P., & Gauld, J. A. (1978). Observations on effects of feeding level on growth and reproduction in haddock, *Melanogrammus aeglefinus* (L.) in captivity. Journal of Fish Biology, 13, 85–98. 10.1111/j.1095-8649.1978.tb03416.x

[jfd13469-bib-0076] Horwood, J. (1993). The Bristol channel sole (*Solea solea* (L.)): A fisheries case study. Advances in Marine Biology, 29, 215–367. 10.1016/S0065-2881(08)60132-7

[jfd13469-bib-0077] Horwood, J. W., Greer Walker, M., & Witthames, P. (1989). The effect of feeding levels on the fecundity of plaice (*Pleuronectes platessa*). Journal of the Marine Biological Association of the United Kingdom, 69, 81–92. 10.1017/S0025315400049122

[jfd13469-bib-0078] Hsu, S. Y., & Hsueh, A. J. W. (2000). Tissue‐specific Bcl‐2 protein partners in apoptosis: An ovarian paradigm. Physiological Reviews, 80, 593–614. 10.1152/physrev.2000.80.2.593 10747202

[jfd13469-bib-0079] Hughes, F. M., & Gorospe, W. C. (1991). Biochemical identification of apoptosis (programmed cell death) in granulosa cells: Evidence for a potential mechanism underlying follicular atresia. Endocrinology, 129, 2415–2422. 10.1210/endo-129-5-2415 1935775

[jfd13469-bib-0080] Hunter, J. R., & Macewicz, B. J. (1985). Rates of atresia in the ovary of captive and wild northern anchovy, *Engraulis mordax* . Fishery Bulletin, 83, 119–136.

[jfd13469-bib-0081] Hunter, J. R., Macewicz, B. J., Lo, N. C. H., & Kimbrell, C. A. (1992). Fecundity, spawning and maturity of female Dover Sole, *Microstomus pacificus*, with an evaluation of assumptions and precision. Fishery Bulletin, 90, 101–128.

[jfd13469-bib-0082] Hunter, J. R., Macewicz, B. J., & Sibert, J. R. (1986). The spawning frequency of skipjack tuna, *Katsuwonus pelamis*, from the South Pacific. Fishery Bulletin, 48, 895–903.

[jfd13469-bib-0083] Hussein, M. R. (2005). Apoptosis in the ovary: Molecular mechanisms. Human Reproduction Update, 11, 162–178. 10.1093/humupd/dmi001 15705959

[jfd13469-bib-0084] Janz, D. M., McMaster, M. E., Munkittrick, K. R., & Van Der Kraak, G. (1997). Elevated ovarian follicular apoptosis and heat shock protein‐70 expression in white sucker exposed to bleached kraft pulp mill effluent. Toxicology and Applied Pharmacology, 147, 391–398. 10.1006/taap.1997.8283 9439734

[jfd13469-bib-0085] Janz, D. M., & Van Der Kraak, G. (1997). Suppression of apoptosis by gonadotropin, 17β‐estradiol, and epidermal growth factor in rain‐bow trout preovulatory ovarian follicles. General and Comparative Endocrinology, 105, 186–193. 10.1006/gcen.1996.6820 9038251

[jfd13469-bib-0086] Jiang, Y.‐X., Shi, W.‐J., Ma, D.‐D., Zhang, J.‐N., Ying, G.‐G., Zhang, H., & Ong, C.‐N. (2019). Dydrogesterone exposure induces zebrafish ovulation but leads to oocytes over‐ripening: An integrated histological and metabolomics study. Environment International, 128, 390–398. 10.1016/j.envint.2019.04.059 31078873

[jfd13469-bib-0087] Jobling, S., Beresford, N., Nolan, M., Rodgers‐Gray, T., Brighty, G. C., Sumpter, J. P., & Tyler, C. R. (2002). Altered sexual maturation and gamete production in wild roach (*Rutilus rutilus*) living in rivers that receive treated sewage effluents. Biology of Reproduction, 66, 272–281. 10.1095/biolreprod66.2.272 11804939

[jfd13469-bib-0088] Johnson, L. L., Sol, S. Y., Ylitalo, G. M., Hom, T., French, B., Olson, O. P., & Collier, T. K. (1999). Reproductive injury in English Sole (*Pleuronectes vetulus*) from the Hylebos Waterway, Commencement Bay, Washington. Journal of Aquatic Ecosystem Stress and Recovery, 6, 289–310. 10.1023/A:1009986329935

[jfd13469-bib-0089] Jørgensen, C., Ernande, B., Fiksen, Ø., & Dieckmann, U. (2006). The logic of skipped spawning in fish. Canadian Journal of Fisheries and Aquatic Sciences, 63, 200–211. 10.1139/f05-210

[jfd13469-bib-0090] Kaptaner, B., & Ünal, G. (2011). Effects of 17α‐ethynylestradiol and nonylphenol on liver and gonadal apoptosis and histopathology in *Chalcalburnus tarichi* . Environmental Toxicology, 26, 610–622. 10.1002/tox.20585 20549615

[jfd13469-bib-0091] Kazanskii, B. N. (1963). Obtaining of different season progeny of fishes for repeated culture cycles (sturgeon as an example). In S. N. Pavlovskii (Ed.), Sturgeon culture in water bodies of the USSR (pp. 56–64). Izdatelstvo Akad Nauk SSSR.

[jfd13469-bib-0092] Kennedy, J., Witthames, P. R., Nashk, R. D. M., & Fox, C. J. (2008). Is fecundity in plaice (*Pleuronectes platessa* L.) down‐regulated in response to reduced food intake during autumn? Journal of Fish Biology, 72, 78–92. 10.1111/j.1095-8649.2007.01651.x

[jfd13469-bib-0093] King Heiden, T., Carvan, M. J. III, & Hutz, R. J. (2006). Inhibition of follicular development, vitellogenesis, and serum 17β‐estradiol concentrations in zebrafish following chronic, sublethal dietary exposure to 2,3,7,8‐Tetrachlorodibenzo‐p‐Dioxin. Toxicological Sciences, 90, 490–499. 10.1093/toxsci/kfj085 16387744

[jfd13469-bib-0094] Kiparissis, Y., Metcalfe, T. L., Balch, G. C., & Metcalfe, C. D. (2003). Effects of the antiandrogens, vinclozolin and cyproterone acetate on gonadal development in the Japanese medaka (*Oryzias latipes*). Aquatic Toxicology, 63, 391–403. 10.1016/S0166-445X(02)00189-3 12758004

[jfd13469-bib-0095] Kjesbu, O. S., Klungsøyr, J., Kryvi, H., Witthames, P. R., & Greer Walker, M. (1991). Fecundity, atresia, and egg size of captive Atlantic cod (*Gadus morhua*) in relation to proximate body composition. Canadian Journal of Fisheries and Aquatic Sciences, 48, 2333–2343. 10.1139/f91-274

[jfd13469-bib-0096] Koger, C. S., The, S. J., & Hinton, D. E. (1999). Variations of light and temperature regimes and resulting effects on reproductive parameters in medaka (*Oryzias latipes*). Biology of Reproduction, 61, 1287–1293. 10.1095/biolreprod61.5.1287 10529276

[jfd13469-bib-0097] Kokokiris, L., Bruslé, S., Kentouri, M., & Fostier, A. (1999). Sexual maturity and hermaphroditism of the red porgy *Pagrus pagrus* (Teleostei: Sparidae). Marine Biology, 134, 621–629. 10.1007/s002270050577

[jfd13469-bib-0098] Kraus, G., Tomkiewicz, J., Diekmann, R., & Köster, F. W. (2008). Seasonal prevalence and intensity of follicular atresia in Baltic cod *Gadus morhua callarias* L. Journal of Fish Biology, 72, 831–847. 10.1111/j.1095-8649.2007.01760.x

[jfd13469-bib-0099] Krysko, D. V., Diez‐Fraile, A., Criel, G., Svistunov, A. A., Vandenabeele, P., & D’Herde, K. (2008). Life and death of female gametes during oogenesis and folliculogenesis. Apoptosis, 13, 1065–1087. 10.1007/s10495-008-0238-1 18622770

[jfd13469-bib-0100] Kurita, Y., Meier, S., & Kjesbu, O. S. (2003). Oocyte growth and fecundity regulation by atresia of Atlantic herring (*Clupea harengus*) in relation to body condition throughout the maturation cycle. Journal of Sea Research, 49, 203–219. 10.1016/S1385-1101(03)00004-2

[jfd13469-bib-0101] Lance, V. A., Rostal, D. C., Elsey, R. M., & Trosclair, P. L. III (2009). Ultrasonography of reproductive structures and hormonal correlates of follicular development in female American alligators, *Alligator mississippiensis*, in southwest Louisiana. General and Comparative Endocrinology, 162, 251–256. 10.1016/j.ygcen.2009.03.021 19344723

[jfd13469-bib-0102] Lethimonier, C., Flouriot, G., Valotaire, Y., Kah, O., & Ducouret, B. (2000). Transcriptional interference between glucocorticoid receptor and estradiol receptor mediates the inhibitory effect of cortisol on fish vitellogenesis. Biology of Reproduction, 62, 1763–1771. 10.1095/biolreprod62.6.1763 10819781

[jfd13469-bib-0103] Li, X., Johnson, R. W., Park, D., Chin‐Sang, I., & Chamberlin, H. M. (2012). Somatic gonad sheath cells and Eph receptor signaling promote germ‐cell death in C. elegans. Cell Death & Differentiation, 19, 1080–1089. 10.1038/cdd.2011.192 22240896 PMC3354057

[jfd13469-bib-0104] Linares‐Casenave, J., Van Eenennaam, J. P., & Doroshov, S. I. (2002). Ultrastructural and histological observations on temperature‐induced follicular ovarian atresia in the white sturgeon. Journal of Applied Ichthyology, 18, 382–390. 10.1046/j.1439-0426.2002.00369.x

[jfd13469-bib-0105] Livingston, M. E., Vignaux, M., & Schofield, K. A. (1997). Estimating the annual proportion of nonspawning adults in New Zealand hoki, *Macruronus novaezelandiae* . Fishery Bulletin, 95, 99–113.

[jfd13469-bib-0106] Lukšienė, D., Sandström, O., Lounasheimo, L., & Andersson, J. (2000). The effects of thermal effluent exposure on the gametogenesis of female fish. Journal of Fish Biology, 56, 37–50. 10.1111/j.1095-8649.2000.tb02085.x

[jfd13469-bib-0107] Ma, Y., Kjesbu, O. S., & Jørgensen, T. (1998). Effects of ration on the maturation and fecundity in captive Atlantic herring (*Clupea harengus*). Canadian Journal of Fisheries and Aquatic Sciences, 55, 900–908. 10.1139/f97-305

[jfd13469-bib-0108] Madureira, T. V., Rocha, M. J., Cruzeiro, C., Galante, M. H., Monteiro, R. A. F., & Rocha, E. (2011). The toxicity potential of pharmaceuticals found in the Douro River estuary (Portugal): Assessing impacts on gonadal maturation with a histopathological and stereological study of zebrafish ovary and testis after sub‐acute exposures. Aquatic Toxicology, 105, 292–299. 10.1016/j.aquatox.2011.06.017 21781654

[jfd13469-bib-0109] Mañanos, E., Duncan, N., & Mylonas, C. C. (2009). Reproduction and control of ovulation, spermiation and spawning in cultured fish. In E. Cabrita, V. Robles, & P. Herraez (Eds.), Methods in reproductive aquaculture (pp. 3–80). CRC Press Taylor and Francis Group.

[jfd13469-bib-0110] Mandich, A., Bottero, S., Benfenati, E., Cevasco, A., Erratico, C., Maggioni, S., Massari, A., Pedemonte, F., & Viganò, L. (2007). In vivo exposure of carp to graded concentrations of bisphenol A. General and Comparative Endocrinology, 153, 15–24. 10.1016/j.ygcen.2007.01.004 17320878

[jfd13469-bib-0111] Matova, N., & Cooley, L. (2001). Comparative aspects of animal oogenesis. Developmental Biology, 231, 291–320. 10.1006/dbio.2000.0120 11237461

[jfd13469-bib-0112] Mayon, N., Bertrand, A., Leroy, D., Malbrouck, C., Mandiki, S. N. M., Silvestre, F., Goffart, A., Thomé, J. P., & Kestemont, P. (2006). Multiscale approach of fish responses to different types of environmental contaminations: A case study. Science of the Total Environment, 367, 715–731. 10.1016/j.scitotenv.2006.03.005 16740295

[jfd13469-bib-0113] Mccully Phillips, S. R., & Ellis, J. R. (2015). Reproductive characteristics and life‐history relationships of starry smooth‐hound *Mustelus asterias* in British waters. Journal of Fish Biology, 87, 1411–1433. 10.1111/jfb.12826 26709214

[jfd13469-bib-0114] McKinney, J. D., & Waller, C. L. (1994). Polychlorinated biphenyls as hormonally active structural analogues. Environmental Health Perspectives, 102, 290–297. 10.1289/ehp.94102290 8033869 PMC1567120

[jfd13469-bib-0115] Medina, A. (2020). Reproduction of Atlantic bluefin tuna. Fish and Fisheries, 21, 1109–1119. 10.1111/faf.12489

[jfd13469-bib-0116] Medina, A., Magro, A., Paullada‐Salmerón, J. A., & Varela, J. L. (2021). An autofluorescence‐based survey of late follicular atresia in ovaries of a teleost fish (*Thunnus thynnus*). Journal of Fish Biology, in press. 10.1111/jfb.14756 33861470

[jfd13469-bib-0117] Metzstein, M. M., Stanfield, G. M., & Horvitz, H. R. (1998). Genetics of programmed cell death in *C. elegans*: Past, present and future. Trends in Genetics, 14, 410–416. 10.1016/S0168-9525(98)01573-X 9820030

[jfd13469-bib-0118] Miles‐Richardson, S. R., Kramer, V. J., Fitzgerald, S. D., Render, J. A., Yamini, B., Barbee, S. J., & Giesy, J. P. (1999). Effects of waterborne exposure of 17 b‐estradiol on secondary sex characteristics and gonads of fathead minnows (*Pimephales promelas*). Aquatic Toxicology, 47, 129–145. 10.1016/S0166-445X(99)00009-0 10092426

[jfd13469-bib-0119] Mion, M., Thorsen, A., Vitale, F., Dierking, J., Herrmann, J. P., Huwer, B., von Dewitz, B. , & Casini, M. (2018). Effect of fish length and nutritional condition on the fecundity of distressed Atlantic cod *Gadus morhua* from the Baltic Sea. Journal of Fish Biology, 92, 1016–1034. 10.1111/jfb.13563 29479694

[jfd13469-bib-0120] Miranda, A. C. L., Bazzoli, N., Rizzo, E., & Sato, Y. (1999). Ovarian follicular atresia in two teleost species: A histological and ultrastructural study. Tissue and Cell, 31, 480–488. 10.1054/tice.1999.0045 18627868

[jfd13469-bib-0121] Mishra, A. K., & Mohanty, B. (2008). Histopathological effects of hexavalent chromium in the ovary of a fresh water fish, *Channa punctatus* (Bloch). Bulletin of Environmental Contamination and Toxicology, 80, 507–511. 10.1007/s00128-008-9406-9 18392725

[jfd13469-bib-0122] Mishra, A. K., & Mohanty, B. (2012). Effect of sublethal hexavalent chromium exposure on the pituitary–ovarian axis of a teleost, *Channa punctatus* (Bloch). Environmental Toxicology, 27, 415–422. 10.1002/tox.20654 22707220

[jfd13469-bib-0123] Mishra, A. K., & Mohanty, B. (2014). Acute spill‐mimicking exposure effect of hexavalent chromium on the pituitary‐ovarian axis of a teleost, *Channa punctatus* (Bloch). Environmental Toxicology, 29, 733–739. 10.1002/tox.21799 22847992

[jfd13469-bib-0124] Mohapatra, S., Kumar, R., Patnaik, S. T., Mishra, C. S., Sahoo, L., & Sundaray, J. K. (2020). Changes in ovary and testis and breeding fitness of the climbing perch, *Anabas testudineus* (Bloch, 1792), exposed to sub‐lethal concentrations of monocrotophos. Aquaculture Research, 51, 3230–3236. 10.1111/are.14657

[jfd13469-bib-0125] Molina, A. M., Abril, N., Morales‐Prieto, N., Monterde, J. G., Lora, A. J., Ayala, N., & Moyano, R. (2018). Evaluation of toxicological endpoints in female zebrafish after bisphenol A exposure. Food and Chemical Toxicology, 112, 19–25. 10.1016/j.fct.2017.12.026 29258955

[jfd13469-bib-0126] Molina, A. M., Lora, A. J., Blanco, A., Monterde, J. G., Ayala, N., & Moyano, R. (2013). Endocrine‐active compound evaluation: Qualitative and quantitative histomorphological assessment of zebrafish gonads after bisphenol‐A exposure. Ecotoxicology and Environmental Safety, 88, 155–162. 10.1016/j.ecoenv.2012.11.010 23219663

[jfd13469-bib-0127] Moodley, G. K., & van Wyk, J. H. (2007). Folliculogenesis and ovarian histology of the oviparous gecko, *Hemidactylus mabouia* (Sauria: Gekkonidae). African Journal of Herpetology, 56, 115–135. 10.1080/21564574.2007.9635558

[jfd13469-bib-0128] Morais, R. D. V. S., Thomé, R. G., Lemos, F. S., Bazzoli, N., & Rizzo, E. (2012). Autophagy and apoptosis interplay during follicular atresia in fish ovary: A morphological and immunocytochemical study. Cell and Tissue Research, 347, 467–478. 10.1007/s00441-012-1327-6 22314847

[jfd13469-bib-0129] Muñoz, M., Dimitriadis, C., Casadevall, M., Vila, S., Delgado, E., Lloret, J., & Saborido‐Rey, F. (2010). Female reproductive biology of the bluemouth *Helicolenus dactylopterus dactylopterus*: Spawning and fecundity. Journal of Fish Biology, 77, 2423–2442. 10.1111/j.1095-8649.2010.02835.x 21155792

[jfd13469-bib-0130] Murua, H., Krous, G., Saborido‐Rey, F., Whittames, P. R., Thorsen, A., & Junquera, S. (2003). Procedures to estimate fecundity in marine fish species in relation to their reproductive strategy. Journal of Northwest Atlantic Fisheries Science, 33, 33–54. 10.2960/J.v33.a3

[jfd13469-bib-0131] Mylonas, C. C., Bridges, C. R., Gordin, H., Belmonte Ríos, A., García, A., de la Gándara, F. , Fauvel, C., Suquet, M., Medina, A., Papadaki, M., Heinisch, G., De Metrio, G., Corriero, A., Vassallo‐Agius, R., Guzmán, J. M., Mañanos, E., & Zohar, Y. (2007). Preparation and administration of gonadotropin‐releasing hormone agonist (GnRHa) implants for the artificial control of reproductive maturation in captive‐reared Atlantic bluefin tuna (*Thunnus thynnus*). Reviews in Fisheries Science, 15, 183–210. 10.1080/10641260701484572

[jfd13469-bib-0132] Mylonas, C. C., Duncan, N. J., & Asturiano, J. F. (2017). Hormonal manipulations for the enhancement of sperm production in cultured fish and evaluation of sperm quality. Aquaculture, 472, 21–44. 10.1016/j.aquaculture.2016.04.021

[jfd13469-bib-0133] Mylonas, C. C., Fostier, A., & Zanuy, S. (2010). Broodstock management and hormonal manipulations of fish reproduction. General and Comparative Endocrinology, 165(516), 534. 10.1016/j.ygcen.2009.03.007 19318108

[jfd13469-bib-0134] Mylonas, C. C., Mitrizakis, N., Castaldo, C. A., Cerviño, C. P., Papadaki, M., & Sigelaki, I. (2013). Reproduction of hatchery‐produced meagre *Argyrosomus regius* in captivity II. Hormonal induction of spawning and monitoring of spawning kinetics, egg production and egg quality. Aquaculture, 414–415, 318–327. 10.1016/j.aquaculture.2013.09.008

[jfd13469-bib-0135] Mylonas, C. C., Sigelaki, I., Divanach, P., Mananos, E., Carrillo, M., & Afonso‐Polyviou, A. (2003). Multiple spawning and egg quality of individual European sea bass (*Dicentrarchus labrax*) females after repeated injections of GnRHa. Aquaculture, 221, 605–620. 10.1016/S0044-8486(03)00120-0

[jfd13469-bib-0136] Mylonas, C. C., Woods, L. C. III, Thomas, P., & Zohar, Y. (1998). Endocrine profiles of female striped bass (*Morone saxatilis*) in captivity, during post‐vitellogenesis and induction of final oocyte maturation via controlled release GnRHa‐delivery systems. General and Comparative Endocrinology, 110, 276–289. 10.1006/gcen.1998.7073 9593648

[jfd13469-bib-0137] Mylonas, C. C., Woods, L. C. III, & Zohar, Y. (1997). Cyto‐histological examination of post‐vitellogenesis and final oocyte maturation in captive‐reared striped bass. Journal of Fish Biology, 50, 34–49. 10.1111/j.1095-8649.1997.tb01338.x

[jfd13469-bib-0138] Mylonas, C. C., Zohar, Y., Pankhurst, N. W., & Kagawa, H. (2011). Reproduction and broodstock management. In M. Pavlidis, & C. C. Mylonas (Eds.), Sparidae: Biology and aquaculture of gilthead seabream and related species (pp. 95–131). Blackwell Science Publishers.

[jfd13469-bib-0139] Neves, A., Vieira, A. R., Farias, I., Figueiredo, I., Sequeira, V., & Gordo, L. S. (2009). Reproductive strategies in black scabbardfish (*Aphanopus carbo* Lowe, 1839) from the NE Atlantic. Scientia Marina, 73S2, 19–31. 10.3989/scimar.2009.73s2019

[jfd13469-bib-0140] Nissling, A., Thorsen, A., & da Silva, F. F. G. (2016). Fecundity regulation by atresia in turbot *Scophthalmus maximus* in the Baltic Sea. Journal of Fish Biology, 88, 1301–1320. 10.1111/jfb.12879 26928526

[jfd13469-bib-0141] Ondricek, K., & Thomas, P. (2018). Effects of hypoxia exposure on apoptosis and expression of membrane steroid receptors, ZIP9, mPRα, and GPER in Atlantic croaker ovaries. Comparative Biochemistry and Physiology Part A, 224, 84–92. 10.1016/j.cbpa.2018.07.002 30025815

[jfd13469-bib-0142] Óskarsson, G. J., Kjesbu, O. S., & Slotte, A. (2002). Predictions of realised fecundity and spawning time in Norwegian spring‐spawning herring (*Clupea harengus*). Journal of Sea Research, 48, 59–79. 10.1016/S1385-1101(02)00135-1

[jfd13469-bib-0143] Pankhurst, N. W., & Van Der Kraak, G. (1997). Effects of stress on reproduction and growth of fish. In G. K. Iwama, J. Sumpter, A. D. Pickering, & C. B. Schreck (Eds.), Fish stress and health in aquaculture (pp. 73–93). Cambridge Univ. Press.

[jfd13469-bib-0144] Pankhurst, N. W., & Van Der Kraak, G. (2000). Evidence that acute stress inhibits ovarian steroidogenesis in rainbow trout *in vivo*, through the action of cortisol. General and Comparative Endocrinology, 117, 225–237. 10.1006/gcen.1999.7401 10642445

[jfd13469-bib-0145] Papadaki, M., Peleteiro, J. B., Alvarez‐Blázquez, B., Rodríguez Villanueva, J. L., Linares, F., Vilar, A., Pérez Rial, E., Lluch, N., Fakriadis, I., Sigelaki, I., & Mylonas, C. C. (2018). Description of the annual reproductive cycle of wreckfish *Polyprion americanus* in captivity. Fishes, 3, 1–20. 10.3390/fishes3040043 29683143

[jfd13469-bib-0146] Passantino, L., Zupa, R., Pousis, C., Mylonas, C. C., Hala, E., Jirillo, E., & Corriero, A. (2020). Increased melanomacrophage centres in the liver of reproductively dysfunctional female greater amberjack *Seriola dumerili* (Risso, 1810). Journal of Fish Diseases, 43, 503–514. 10.1111/jfd.13149 32103518

[jfd13469-bib-0147] Patiño, R., & Sullivan, C. V. (2002). Ovarian follicle growth, maturation, and ovulation in teleost fish. Fish Physiology and Biochemistry, 26, 57–70. 10.1023/A:1023311613987

[jfd13469-bib-0148] Pérez, N., & Figueiredo, I. (1992). First approach to the study of atresia in the ovary of sardine, *Sardina pilchardus* (Walb.). Boletin Del Instituto Espanol De Oceanografia, 8, 191–199.

[jfd13469-bib-0149] Perini, V. R., Paschoalini, A. L., Cruz, C. K. F., Rocha, R. C. G. A., Senhorini, J. A., Ribeiro, D. M., Formagio, P. S., Bazzoli, N., & Rizzo, E. (2013). Profiles of sex steroids, fecundity and spawning of a migratory characiform fish from the Paraguay‐Paraná basin: A comparative study in a three‐river system. Fish Physiology and Biochemistry, 39, 1473–1484. 10.1007/s10695-013-9800-z 23616136

[jfd13469-bib-0150] Pfuderer, P., Williams, P., & Francis, A. A. (1974). Partial purification of the crowding factor from *Carassius auratus* and *Cyprinus carpio* . Journal of Experimental Zoology, 187, 375–382. 10.1002/jez.1401870306 4362349

[jfd13469-bib-0151] Piccinno, M., Zupa, R., Corriero, A., Centoducati, G., Passantino, L., Rizzo, A., & Sciorsci, R. L. (2014). In vitro effect of isotocin on ovarian tunica albuginea contractility of gilthead seabream (*Sparus aurata* L.) in different reproductive conditions. Fish Physiology and Biochemistry, 40, 1191–1199. 10.1007/s10695-014-9915-x 24482096

[jfd13469-bib-0152] Pickering, A. D., Pottinger, T. G., & Sumpter, J. P. (1987). On the use of dexamethasone to block the pituitary‐interrenal axis in the brown trout, *Salmo trutta* L. General and Comparative Endocrinology, 65, 346–353. 10.1016/0016-6480(87)90119-5 3030876

[jfd13469-bib-0153] Pittman, R. C., & Steinherg, D. (1984). Sites and mechanisms of uptake and degradation of high density and low density lipoproteins. Journal of Lipid Research, 25, 1577–1585. 10.1016/S0022-2275(20)34435-7 6397563

[jfd13469-bib-0154] Plaza, G., Sakaji, H., Honda, H., Hirota, Y., & Nashida, K. (2007). Spawning pattern and type of fecundity in relation to ovarian allometry in the round herring *Etrumeus teres* . Marine Biology, 152, 1051–1064. 10.1007/s00227-007-0756-3

[jfd13469-bib-0155] Porter, M. J. R., Woolcott, H. M., & Pankhurst, N. W. (2003). The use of additional lighting and artificial photoperiods to recondition early maturing Atlantic salmon (*Salmo salar*) in Tasmania. Fish Physiology and Biochemistry, 28, 391–393. 10.1023/B:FISH.0000030603.30648.90

[jfd13469-bib-0156] Pousis, C., Mylonas, C. C., De Virgilio, C., Gadaleta, G., Santamaria, N., Passantino, L., Zupa, R., Papadaki, M., Fakriadis, I., Ferreri, R., & Corriero, A. (2018). The observed oogenesis impairment in greater amberjack *Seriola dumerili* (Risso, 1810) reared in captivity is not related to an insufficient liver transcription or oocyte uptake of vitellogenin. Aquaculture Research, 49, 243–252. 10.1111/are.13453

[jfd13469-bib-0157] Pousis, C., Rodríguez, C., De Ruvo, P., De Virgilio, C., Pérez, J., Mylonas, C. C., Zupa, R., Passantino, L., Santamaria, N., Valentini, L., & Corriero, A. (2019). Vitellogenin receptor and fatty acid profiles of individual lipid 1 classes of oocytes from wild and captive‐reared greater amberjack (*Seriola dumerili*) during the reproductive cycle. Theriogenology, 140, 73–83. 10.1016/j.theriogenology.2019.08.014 31465910

[jfd13469-bib-0158] Pousis, C., Santamaria, N., Zupa, R., De Giorgi, C., Mylonas, C. C., Bridges, C. R., de la Gándara, F. , Vassallo‐Agius, R., Bello, G., & Corriero, A. (2012). Expression of vitellogenin receptor gene in the ovary of wild and captive Atlantic bluefin tuna (*Thunnus thynnus*). Animal Reproduction Science, 132, 101–110. 10.1016/j.anireprosci.2012.03.014 22541277

[jfd13469-bib-0159] Prado, P. S., Pinheiro, A. P. B., Bazzoli, N., & Rizzo, E. (2014). Reproductive biomarkers responses induced by xenoestrogens in the characid fish *Astyanax fasciatus* inhabiting a South American reservoir: An integrated field and laboratory approach. Environmental Research, 131, 165–173. 10.1016/j.envres.2014.03.002 24721135

[jfd13469-bib-0160] Quirk, S. M., Cowan, R. G., Harman, R. M., Hu, C.‐L., & Porter, D. A. (2004). Ovarian follicular growth and atresia: The relationship between cell proliferation and survival Ovarian follicular growth and atresia: The relationship between cell proliferation and survival. Journal of Animal Science, 82, 40–52. 10.2527/2004.8213_supplE40x 15471814

[jfd13469-bib-0161] Ramsay, J. M., Feist, G. W., Varga, Z. M., Westerfield, M., Kent, M. L., & Schreck, C. B. (2006). Whole‐body cortisol is an indicator of crowding stress in adult zebrafish, *Danio rerio* . Aquaculture, 258, 565–574. 10.1016/j.aquaculture.2006.04.020

[jfd13469-bib-0162] Renuka, K., & Joshi, B. N. (2010). Melatonin‐induced changes in ovarian function in the freshwater fish *Channa punctatus* (Bloch) held in long days and continuous light. General and Comparative Endocrinology, 165, 42–46. 10.1016/j.ygcen.2009.05.020 19501093

[jfd13469-bib-0164] Rideout, R. M., Burton, M. P. M., & Rose, G. A. (2000). Observations on mass atresia and skipped spawning in northern Atlantic cod, from Smith Sound, Newfoundland. Journal of Fish Biology, 57, 1429–1440. 10.1111/j.1095-8649.2000.tb02222.x

[jfd13469-bib-0165] Rideout, R. M., & Rose, G. A. (2006). Suppression of reproduction in Atlantic cod (*Gadus morhua* L.). Marine Ecology Progress Series, 320, 267–277. 10.3354/meps320267

[jfd13469-bib-0166] Rideout, R. M., Rose, G. A., & Burton, M. P. M. (2005). Skipped spawning in female iteroparous fishes. Fish and Fisheries, 6, 50–72. 10.1111/j.1467-2679.2005.00174.x

[jfd13469-bib-0167] Rideout, R. M., & Tomkiewicz, J. (2011). Skipped spawning in fishes: More common than you might think. Marine and Coastal Fisheries: Dynamics, Management, and Ecosystem Science, 3, 176–189. 10.1080/19425120.2011.556943

[jfd13469-bib-0168] Rosenfeld, H., Mylonas, C. C., Bridges, C. R., Heinisch, G., Corriero, A., Vassallo‐Agius, R., Medina, A., Belmonte, A., Garcia, A., de la Gándara, F. , Fauvel, C., De Metrio, G., Meiri‐Ashkenazi, I., Gordin, H., & Zohar, Y. (2012). GnRHa‐mediated stimulation of the reproductive endocrine axis in captive Atlantic bluefin tuna, *Thunnus thynnus* . General and Comparative Endocrinology, 175, 55–64. 10.1016/j.ygcen.2011.09.013 22015989

[jfd13469-bib-0169] Roy, R. L., Ruby, S. M., Idler, D. R., & So, Y. (1990). Plasma vitellogenin levels in pre‐spawning rainbow trout, *Oncorhynchus mykiss*, during acid exposure. Archives of Environmental Contamination and Toxicology, 19, 803–806. 10.1007/BF01055044

[jfd13469-bib-0170] Saidapur, S. K. (1978). Follicular atresia in the ovaries of nonmammalian vertebrates. International Review of Cytology, 54, 225–244. 10.1016/S0074-7696(08)60169-2 391757

[jfd13469-bib-0171] Santos, H. B., Thomé, R. G., Arantes, F. P., Sato, Y., Bazzoli, N., & Rizzo, E. (2008). Ovarian follicular atresia is mediated by heterophagy, autophagy, and apoptosis in *Prochilodus argenteus* and *Leporinus taeniatus* (Teleostei: Characiformes). Theriogenology, 70, 1449–1460. 10.1016/j.theriogenology.2008.06.091 18701155

[jfd13469-bib-0172] Sato, Y., Bazzoli, N., Rizzo, E., Boschi, M. B., & Miranda, M. O. T. (2005). Influence of the Abaeté River on the reproductive success of neotropical migratory teleost *Prochilodus argenteus* in the São Francisco River, downstream from the Três Marias Dam, southeastern Brazil. River Research and Applications, 21, 939–950. 10.1002/rra.859

[jfd13469-bib-0173] Sayed, A. H., Ismail, R. F., & Mitani, H. (2018). Oocyte atresia in WT (HdrR) and P53 (‐/‐) medaka (*Oryzias latipes*) exposed to UVA. Journal of Photochemistry & Photobiology, B: Biology, 183, 57–63. 10.1016/j.jphotobiol.2018.04.016 29684721

[jfd13469-bib-0174] Schreck, C. B. (2010). Stress and fish reproduction: The roles of allostasis and hormesis. General and Comparative Endocrinology, 165, 549–556. 10.1016/j.ygcen.2009.07.004 19596332

[jfd13469-bib-0175] Schreck, C. B., Contreras‐Sanchez, W., & Fitzpatrick, M. S. (2001). Effects of stress on fish reproduction, gamete quality, and progeny. Aquaculture, 197, 3–24. 10.1016/S0044-8486(01)00580-4

[jfd13469-bib-0176] Schulz, R., & Blüm, V. (1983). Elimination of the nucleus in preovulatory oocytes of the rainbow trout, *Salmo gairdneri* Richardson (Teleostei). Cell and Tissue Research, 232, 685–689. 10.1007/BF00216439 6883465

[jfd13469-bib-0177] Scott, D. P. (1962). Effect of food quantity on fecundity of rainbow trout, *Salmo gairdneri* . Journal of the Fisheries Research Board of Canada, 19, 715–731. 10.1139/f62-047

[jfd13469-bib-0178] Sepulcre, M. P., Pelegrín, P., Mulero, V., & Meseguer, J. (2002). Characterisation of gilthead seabream acidophilic granulocytes by a monoclonal antibody unequivocally points to their involvement in fish phagocytic response. Cell and Tissue Research, 308, 97–102. 10.1007/s00441-002-0531-1 12012209

[jfd13469-bib-0179] Skjæraasen, J. E., Korsbrekke, K., Kjesbub, O. S., Fonn, M., Nilsen, T., & Nash, R. D. M. (2013). Size‐, energy‐ and stage‐dependent fecundity and the occurrence of atresia in the Northeast Arctic haddock *Melanogrammus aeglefinus* . Fisheries Research, 138, 120–127. 10.1016/j.fishres.2012.04.003

[jfd13469-bib-0180] Sridevi, P., Chaitanya, R. K., Prathibha, Y., Balakrishna, S. L., Dutta‐Gupta, A., & Senthilkumaran, B. (2015). Early Exposure of 17α‐ethynylestradiol and diethylstilbestrol induces morphological changes and alters ovarian steroidogenic pathway enzyme gene expression in catfish, *Clarias gariepinus* . Environmental Toxicology, 30, 439–451. 10.1002/tox.21920 24273110

[jfd13469-bib-0181] Steller, H. (1995). Mechanisms and genes of cellular suicide. Science, 267, 1445–1449. 10.1126/science.7878463 7878463

[jfd13469-bib-0182] Sumpter, J. P., Le Bail, P. Y., Pickering, A. D., Pottinger, T. G., & Carragher, J. F. (1991). The effect of starvation on growth and plasma growth hormone concentrations of rainbow trout, *Oncorhynchus mykiss* . General and Comparative Endocrinology, 83, 94–102. 10.1016/0016-6480(91)90109-J 1879676

[jfd13469-bib-0183] Talbott, M. J., Van Eenennaam, J. P., Linares‐Casenave, J., Doroshov, S. I., Guy, C. S., Struffenegger, P., & Webb, M. A. H. (2011). Investigating the use of plasma testosterone and estradiol‐17β to detect ovarian follicular atresia in farmed white sturgeon, *Acipenser transmontanus* . Aquaculture, 315, 283–289. 10.1016/j.aquaculture.2010.10.041

[jfd13469-bib-0184] Thomas, P., Rahman, M. S., Picha, M. E., & Tan, W. (2005). Impaired gamete production and viability in Atlantic croaker collected throughout the 20,000 km^2^ hypoxic region in the northern Gulf of Mexico. Comparative Biochemistry and Physiology Part A, 224, 84–92. 10.1016/j.marpolbul.2015.11.001 26547103

[jfd13469-bib-0185] Thomé, R. G., Domingos, F. F. T., Santos, H. B., Martinelli, P. M., Sato, Y., Rizzo, E., & Bazzoli, N. (2012). Apoptosis, cell proliferation and vitellogenesis during the folliculogenesis and follicular growth in teleost fish. Tissue and Cell, 44, 54–62. 10.1016/j.tice.2011.11.002 22153985

[jfd13469-bib-0186] Thomé, R. G., Santos, H. B., Arantes, F. P., Domingos, F. F. T., Bazzoli, N., & Rizzo, E. (2009). Dual roles for autophagy during follicular atresia in fish ovary. Autophagy, 5, 117–119. 10.4161/auto.5.1.7302 19011378

[jfd13469-bib-0187] Thorsen, A., Marshall, C. T., & Kjesbu, O. S. (2006). Comparison of various potential fecundity models for North‐east Arctic cod *Gadus morhua*, L. using oocyte diameter as a standardising factor. Journal of Fish Biology, 69, 1709–1730. 10.1111/j.1095-8649.2006.01239.x

[jfd13469-bib-0188] Tilly, J. L. (1996a). Apoptosis and ovarian function. Reviews of Reproduction, 1, 162–172. 10.1530/ror.0.0010162 9414454

[jfd13469-bib-0189] Tilly, J. L. (1996b). The molecular basis of ovarian cell death during germ cell attrition, follicular atresia, and luteolysis. Frontiers in Bioscience, 1, d1–11. 10.2741/a111 9159202

[jfd13469-bib-0190] Tilly, J. L., Kowalski, K. I., Johnson, A. L., & Hsueh, A. J. (1991). Involvement of apoptosis in ovarian follicular atresia and postovulatory regression. Endocrinology, 129, 2799–2801. 10.1210/endo-129-5-2799 1718732

[jfd13469-bib-0191] Tilly, J. L., Tilly, K. I., & Perez, G. I. (1997). The genes of cell death and cellular susceptibility to apoptosis in the ovary: A hypothesis. Cell Death and Differentiation, 43, 180–187. 10.1038/sj.cdd.4400238 16465227

[jfd13469-bib-0192] Tingaud‐Sequeira, A., Chauvigné, F., Lozano, J., Agulleiro, M. J., Asensio, E., & Cerdà, J. (2006). New insights into molecular pathways associated with flatfish ovarian development and atresia revealed by transcriptional analysis. BMC Genomics, 10, 434. 10.1186/1471-2164-10-434 PMC2751788 19754951

[jfd13469-bib-0193] Townson, D. H., & Liptak, A. R. (2003). Chemokines in the corpus luteum: Implications of leukocyte chemotaxis. Reproductive Biology and Endocrinology, 1, 94. 10.1186/1477-7827-1-94 14613530 PMC293429

[jfd13469-bib-0194] Tyler, A. V., & Dunn, R. S. (1976). Ration, growth, and measures of somatic and organ condition in relation to meal frequency in winter flounder, *Pseudopleuronectes americanus*, with hypotheses regarding population homeostasis. Journal of the Fisheries Research Board of Canada, 33, 63–75. 10.1139/f76-008

[jfd13469-bib-0195] Ukeshima, A., & Fujimoto, T. (1991). A fine morphological study of germ cells in asymmetrically developing right and left ovaries of the chick. The Anatomical Record, 230, 378–386. 10.1002/ar.1092300311 1867412

[jfd13469-bib-0197] Vaux, D. L., & Korsmeyer, S. J. (1999). Cell death in development. Cell, 96, 245–254. 10.1016/s0092-8674(00)80564-4 9988219

[jfd13469-bib-0198] Wallace, R. A., & Selman, K. (1981). Cellular and dynamic aspects of oocyte growth in teleosts. American Zoologist, 21, 325–343. 10.1093/icb/21.2.325

[jfd13469-bib-0199] Waltrick, D. S., Simpfendorfer, C. A., & Awruch, C. A. (2017). A review on the morphology of ovarian follicles in elasmobranchs: A case study in *Rhizoprionodon taylori* . Journal of Morphology, 278, 486–499. 10.1002/jmor.20644 28165147

[jfd13469-bib-0200] Webb, M. A. H., Van Eeneennam, J. P., Doroshov, S. I., & Moberg, G. P. (1999). Preliminary observations on the effects of holding temperature on reproductive performance of female white sturgeon, *Acipenser transmontanus* Richardson. Aquaculture, 176, 315–329. 10.1016/S0044-8486(99)00108-8

[jfd13469-bib-0201] Webb, M. A. H., Van Eenennaam, J. P., Feist, G. W., Linares‐Casenave, J., Fitzpatrick, M. S., Schreck, C. B., & Doroshov, S. I. (2001). Effects of thermal regime on ovarian maturation and plasma sex steroids in farmed white sturgeon, *Acipenser transmontanus* . Aquaculture, 201, 137–151. 10.1016/S0044-8486(01)00550-6

[jfd13469-bib-0202] Wendelaar Bonga, B. S. E. (1997). The stress response in fish. Physiological Reviews, 77, 591–625. 10.1152/physrev.1997.77.3.591 9234959

[jfd13469-bib-0203] Wirbisky, S. E., Weber, G. J., Sepúlveda, M. S., Lin, T.‐L., Jannasch, A. S., & Freeman, J. L. (2016). An embryonic atrazine exposure results in reproductive dysfunction in adult zebrafish and morphological alterations in their offspring. Scientific Report, 6, 21337. 10.1038/srep21337 PMC4759560 26891955

[jfd13469-bib-0204] Witthames, P. R., & Greer Walker, M. (1995). Determinacy of fecundity and oocyte atresia in sole (*Solea solea*) from the Channel, the North Sea and the Irish Sea. Aquatic Living Resources, 8, 91–109. 10.1051/alr:1995007

[jfd13469-bib-0205] Wood, A. W., & Van Der Kraak, G. J. (2001). Apoptosis and ovarian function: Novel perspectives from the teleosts. Biology of Reproduction, 64, 264–271. 10.1095/biolreprod64.1.264 11133683

[jfd13469-bib-0206] Wood, A. W., & Van der Kraak, G. (2002). Inhibition of apoptosis in vitellogenic ovarian follicles of rainbow trout (*Oncorhynchus mykiss*) by salmon gonadotropin, epidermal growth factor, and 17beta‐estradiol. Molecular Reproduction & Development, 61, 511–518. 10.1002/mrd.10108 11891923

[jfd13469-bib-0207] Yamamoto, Y. A., Luckenbach, J., Goetz, F. W., Young, G., & Swanson, P. (2011). Disruption of the salmon reproductive endocrine axis through prolonged nutritional stress: Changes in circulating hormone levels and transcripts for ovarian genes involved in steroidogenesis and apoptosis. General and Comparative Endocrinology, 172, 331–343. 10.1016/j.ygcen.2011.03.017 21447335

[jfd13469-bib-0208] Yan, W., Hamid, N., Deng, S., Jia, P.‐P., & Pei, D.‐S. (2020). Individual and combined toxicogenetic effects of microplastics and heavy metals (Cd, Pb, and Zn) perturb gut microbiota homeostasis and gonadal development in marine medaka (*Oryzias melastigma*). Journal of Hazardous Materials, 397, 122795. 10.1016/j.jhazmat.2020.122795 32388101

[jfd13469-bib-0209] Ye, T., Kang, M., Huang, Q., Fang, C., Chen, Y., Shen, H., & Dong, S. (2014). Exposure to DEHP and MEHP from hatching to adulthood causes reproductive dysfunction and endocrine disruption in marine medaka (*Oryzias melastigma*). Aquatic Toxicology, 146, 115–126. 10.1016/j.aquatox.2013.10.025 24292025

[jfd13469-bib-0210] Yoshioka, H. (1962). On the effects of environmental factors upon the reproduction of fishes. 1. The effects of day‐length on the reproduction of the Japanese Killifish, *Oryzias latipes* . Bulletin of the Faculty of Fisheries, Hokkaido University, 13, 123–136.

[jfd13469-bib-0211] Yoshioka, H. (1963). On the effects of environmental factors upon the reproduction of fishes 2. Effects of Short and Long Day‐lengths on *Oryzias latipes* during spawning season. Bulletin of the Faculty of Fisheries, Hokkaido University, 14, 137–151.

[jfd13469-bib-0212] Young, K. A., & Nelson, R. J. (2001). Mediation of seasonal testicular regression by apoptosis. Reproduction, 122, 677–685. 10.1530/rep.0.1220677 11690527

[jfd13469-bib-0213] Zohar, Y. (2021). Fish reproductive biology – Reflecting on five decades of fundamental and translational research. General and Comparative Endocrinology, 300, 113544. 10.1016/j.ygcen.2020.113544 32615136 PMC7324349

[jfd13469-bib-0214] Zohar, Y., Muñoz‐Cueto, J. A., Elizur, A., & Kah, O. (2010). Neuroendocrinology of reproduction in teleost fish. General and Comparative Endocrinology, 165, 438–455. 10.1016/j.ygcen.2009.04.017 19393655

[jfd13469-bib-0215] Zohar, Y., & Mylonas, C. C. (2001). Endocrine manipulations of spawning in cultured fish: From hormones to genes. Aquaculture, 197, 99–136. 10.1016/S0044-8486(01)00584-1

[jfd13469-bib-0216] Zupa, R., Fauvel, C., Mylonas, C. C., Pousis, C., Santamaria, N., Papadaki, Μ., Fakriadis, I., Cicirelli, V., Mangano, S., Passantino, L., Lacalandra, G. M., & Corriero, A. (2017). Rearing in captivity affects spermatogenesis and sperm quality in greater amberjack *Seriola dumerili* (Risso, 1810). Journal of Animal Science, 95, 4085–4100. 10.2527/jas2017.1708 28992003

[jfd13469-bib-0217] Zupa, R., Fauvel, C., Mylonas, C. C., Santamaria, N., Valentini, L., Pousis, C., Papadaki, M., Suquet, M., de la Gándara, F. , Bello, G., De Metrio, G., & Corriero, A. (2013). Comparative analysis of male germ cell proliferation and apoptosis in wild and captive Atlantic bluefin tuna *Thunnus thynnus* . Journal of Applied Ichthyology, 29, 71–81. 10.1111/j.1439-0426.2012.02045.x

[jfd13469-bib-0218] Zupa, R., Rodríguez, C., Mylonas, C. C., Rosenfeld, H., Fakriadis, I., Papadaki, M., Pérez, J. A., Pousis, C., Basilone, G., & Corriero, A. (2017). Comparative study of reproductive development in wild and captive‐reared greater amberjack *Seriola dumerili* (Risso, 1810). PLoS One, 12(1), e0169645. 10.1371/journal.pone.0169645 28056063 PMC5215828

